# Survival analysis of time to reimbursement of novel medicines in five Eurasian countries

**DOI:** 10.1186/s41256-025-00457-3

**Published:** 2025-10-27

**Authors:** Zhitao Wang, Yihan Fu, Jing Sun, Yuanli Liu

**Affiliations:** School of Health Policy and Management, Chinese Academy of Medical Sciences and Peking Union Medical College, Beijing, China

**Keywords:** Novel medicines, Public funding, Time to reimbursement, Survival analysis, Cox proportional hazards regression

## Abstract

**Background:**

Access to novel medications matters quality-adjusted life years and the opportunity cost associated with productivity lost. Gaps in patient access to novel medicines exist due to insufficient public funding reimbursement in emerging countries. Evidence of time from regulatory approval to reimbursement decision by public funding, referred to as time to reimbursement (TTR), remained limited in emerging countries. This study compared and analyzed public funding reimbursement of novel medicines approved in five Eurasian countries that are global leaders in pharmaceutical innovation. All of them have a centralized mechanism for reimbursement decisions on novel medicines, allowing identification of a clear date of public funding reimbursement. By exploring the facilitators of rapid application of pharmaceutical innovations, we expected to inform the public funding reimbursement decision-making in emerging countries, so as to improve patient access and contribute to addressing the global health challenge in achieving universal health coverage.

**Methods:**

This is a retrospective study which investigated the public funding reimbursement and TTR of novel medicines that obtained marketing authorization between 2018 and 2023 in China, Japan, France, the United Kingdom (UK) and Switzerland. We firstly conducted descriptive analyses of TTR across countries, followed by the pairwise comparisons using Kruskal–Wallis *H* tests with Bonferroni corrections. We then performed the survival analysis of time-to-event data using the multiple Cox proportional hazards regression by inclusion of country and year dummy variables. Other covariates associated with the characteristics of novel medicines and manufacturers, as well as the review and approval pathways were included in the regression. We estimated the differences of hazard ratios (HR) of novel medicines being reimbursed by public funding across countries. Subgroup analyses were conducted to assess the specific factors associated with the public funding reimbursement in different countries. Since China began to systematically publicly fund novel medicines in 2019, sensitivity analyses were conducted by removing the 2018 data and repeating the same analyses.

**Results:**

As of July 1st, 2024, Japan had the highest proportion and fastest rate of public funding reimbursement of novel medicines, which were approved between 2018 and 2023 (HR = 11.29, [95% CI 8.63, 14.77], *P* < 0.001). In contrast, the TTR of novel medicines approved in China was generally longer than those in the other four countries. Factors associated with a higher likelihood of being reimbursed by public funding included priority review procedure in China and the UK, medicines for rare diseases approved in Japan and France, anti-cancer medicines approved in the UK, locally developed novel medicines approved in China and Switzerland, and medicines launched by large multinational pharmaceutical companies in France and Switzerland. China was the only country in which novel medicines approved through conditional market authorization were less likely to be publicly funded (HR = 0.42, 95% CI [0.27, 0.68], *P* < 0.001).

**Conclusions:**

Compared to other global pharmaceutical innovation leaders, China still needs to make further efforts in strengthening public funding reimbursement of novel medicines. A forward-thinking strategy for health technology assessment that provides advanced technical support in conjunction with the regulatory authority to pharmaceutical innovation companies at the early research and development stage is critical for reducing TTR of novel medicines and accelerating patient access. To balance timely patient access and risk control, strategies such as risk-sharing mechanisms for novel medicines with clinical and cost uncertainties, and temporary reimbursement with alternative sources of funding to support real-world evidence collection could be considered.

**Supplementary Information:**

The online version contains supplementary material available at 10.1186/s41256-025-00457-3.

## Background

Cutting-edge treatments have been increasingly granted with market authorization (MA) in emerging countries [1]. This trend has been driven by the global expansion of pharmaceutical research and development, as well as regulatory reforms aimed at accelerating the approval of novel medicines [2]. However, despite the growing availability of these therapies, clinically impactful novel medicines remain sparsely eligible for reimbursement by public funding. There exists a substantial gap between regulatory approval and reimbursement by public funding [3]. As time to reimbursement (TTR) of novel medicines directly affects patient access, delays in reimbursement by public funding and inequalities in access to novel medicines leave many patients unable to access life-saving therapies [4]. Rapidly rising prices of novel medicines has been intensifying the fact that only a small number of patients could afford novel medicines [3, 5, 6]. For most patients, reimbursement by public funding that provided moderate compensation was the only possible way to afford these medicines [2]. Delay of reimbursement by public funding would have implications not only in loss of quality-life-years, but also opportunity cost due to productivity lost [7–10]. This situation is particularly pronounced in emerging countries. Unlike high-income countries (HICs) where most novel medicines are publicly funded, most emerging countries have been on the way of improving patient access to novel therapies [11]. The disparity in patient access to novel medications exacerbates global health inequities and undermines efforts to achieve universal health coverage [12].

As an emerging country that increasingly contributes to the global pharmaceutical innovation, China has been in the "second tier" in terms of contribution to the global pipeline of novel medicines development. By 2024, this proportion had risen to 26.7%, just behind 49.1% of the United States (US) [13–15]. While, compared with the other global pharmaceutical innovation leaders, public spending on novel medicines in China is still low [4]. Addressing the gap between booming innovation and delayed patient access requires appropriate public funding reimbursement decisions making on novel medicines, which balance timely patient access and risk control of clinical and cost uncertainties. Exploring the potential factors associated with the length of time from regulatory approval to reimbursement decision making by public payers, refers to as TTR, and understanding how other global pharmaceutical innovation leaders made rapid application of pharmaceutical innovations, will help China and the other emerging countries to optimize their policies to improve patient access to novel medicines.

Decision for reimbursement of novel medicines by public funding is made following evaluation of clinical benefits improvement and price negotiation based on health technology assessment (HTA) in most HICs, either in the private health insurance dominated US, where novel medicines are paid by multiple private insurance funds; or the statutory health insurance dominated systems in China, Japan and most European countries; or the National Health Service (NHS) system in the United Kingdom (UK), where there is a centralized mechanism for reimbursement decision making on novel medicines [4]. Details about the public funding reimbursement decision making on novel medicines in the countries included in this study were summarized in Table S13 of the Supplementary material. Existing studies about TTR of novel medicines mainly target HICs like the US and the European countries, including comparative studies across these countries. Evidence showed that TTRs for novel medicines is influenced by multiple factors and varied significantly across countries, with faster coverage in Germany, France, and Switzerland [16–19]. Theoretically, novel medicines are immediately covered by the statutory health insurance following MA in Germany. 6 months is needed for performing HTA in France and Switzerland. Such time extends to at least 8 months in the UK, and shortens to 2–3 months in Japan [4]. Countries with higher gross domestic product, per capita health expenditure, Health Development Index, and health policy scores (e.g., Germany, France, Switzerland) demonstrate significantly faster reimbursement speed [16–18, 20]. Differences in HTA processes affect TTR. Simplified pre-assessment procedures or flexible economic evaluation requirements may accelerate access, whereas strict HTA standards—requiring comprehensive clinical trial data or additional genomic validation for high-risk or evidence-limited medicines (e.g., orphan medicines or medicines approved through a conditional or special pathways) may prolong TTR [17, 19, 21]. Moreover, medicines and manufacturer characteristics may also affect reimbursement timelines. Oncology orphan medicines and medicines developed by large pharmaceutical companies are more likely to be rapidly reimbursed by public funding [16, 21], whereas medicines with higher market prices tend to experience longer waiting time [17]. While, studies about real-world TTR of novel medicines in emerging countries remains limited, only a few recent studies focused on TTR of anti-cancer medicines in China [22, 23], no comparative study is available. Studies about TTR in China found that locally developed medicines were more likely to be reimbursed, whereas medicines marketed through the conditional approval pathway experienced slower reimbursement compared to those approved through conventional pathway [22]. Moreover, optimization of reimbursement decision making process and institutionalization significantly shortened TTR [23]. Inclusion of emerging countries like China that increasingly contribute to the global pharmaceutical innovation in the international comparative TTR study would help to identify the gaps and inform the decision making of reimbursement of novel medicines by public funding in these settings.

This study targeted the top contributors of the global pharmaceutical innovation [13, 14], which are mainly HICs with rapid application of novel medicines. China was included as a typical emerging pharmaceutical innovation leader but with comparatively insufficient public spending on novel medications. Identifying the factors associated with TTR, and understanding the dynamics of reimbursement of novel medicines by public funding in the other global pharmaceutical innovation leaders, will help to inform the decision-making of reimbursement by public funding of novel medicines in China and the other emerging countries to improve patient access, so as to contribute to addressing the global heath challenge in achieving universal health coverage.

## Methods

### Study design

This is a retrospective study based on analysis of survival data, consisting of the time-to-event outcome with right-censoring. Given that some medicines were not publicly reimbursed until the end of the observation time in respective countries, we performed the survival analysis which included the status of non-reimbursement by public funding as a binary outcome on its own [16, 24]. Through which, the relative risk of novel medicines being reimbursed by public funding in five Eurasian global leaders of pharmaceutical innovation were estimated, and factors associated with reimbursement by public funding and TTR were explored.

To obtain a clear and comparable time point of reimbursement of novel medicines by public funding in a specific country, this study only included the top contributors to global pharmaceutical innovation (the US, the Europe, Japan and China) with a centralized mechanism for reimbursement decision on novel medicines [25]. The US was excluded due to that its public funding only targets specific populations, and there are multiple mechanisms for reimbursement decisions on novel medicines. Among the European countries, Germany, the UK and France are the top 3 economies, and Switzerland was ranked first in the Global Innovation Index 2019 [26]. Considered that novel medicines are immediately reimbursed by its statutory health insurance, Germany was excluded [4]. Five Eurasia countries, including the UK, France, Switzerland, Japan and China were finally selected. According to the 2019 Global Innovation Index, the medical innovation performances of these countries were above the expectations for their levels of development [26]. These countries also have a comparable annual number of novel medicines approved during the observation period. The dates of MA and reimbursement decision by public funding in all countries are publicly available from official sources.

China began to systematically include novel medicines into its national basic health insurance reimbursement list in 2019 [4]. To ensure comparability, we targeted novel medicines approved by the national medicine regulatory authorities of five countries from January 1st, 2018 to December 31st, 2023, and those reimbursed by the public funding by July 1st, 2024. As countries have different definitions of novel medicines (Table S1), this study defined novel medicines as medicines or combinations containing new active ingredients, including chemical and biological products [4, 27].

### Variables

The primary variable is the reimbursement status by public funding of the target novel medicines and TTR in respective countries, which was defined as “1” if the medicines were publicly funded by July 1st, 2024, and defined as “0” if they were not publicly funded. TTR refers to the length of time from regulatory approval to reimbursement decision-making by public funding. Specifically, it is defined as the number of days between the date of granting MA and the date that the decision of reimbursement announced by the statutory health insurance of China, Japan, France and Switzerland, or the date of acknowledged Technology Appraisal Guidance published by the National Institute for Health and Care Excellence (NICE) of the UK.

We selected a set of covariates based on existing literature [16, 22, 28]. As all target countries formulated policies to accelerate the review and approval of novel medicines, such as priority review (PR) procedure and conditional market authorization (CMA), especially for the products with significant public health significance, these accelerated review and approval procedures might be associated with the reimbursement decision of public funding. The characteristics of novel medicines were also important potential factors that might affect TTR, and were included as covariates as well, such as if the target medicines are for rare diseases (RDs) or cancers as they are often developed and approved more rapidly worldwide due to their high unmet medical needs, which may further shape their TTR [28]. In addition, we are interested in whether local development would gain support from the public funding reimbursement decision making, and if manufacturing company is based in the country where MA is granted, as well as if the size of the manufacturing company affect the reimbursement decision of public funding and TTR. Country and year dummies were included in the Cox regression model to adjust the inherent time-invariant characteristics of different countries, such as healthcare systems and reimbursement decision making process for public funding, etc., and to mitigate bias from unobserved time-specific confounders.

### Data source

Dates of MA and reimbursement decision of public funding were obtained from the national medicines regulatory authorities of respective countries [29–43] and their official websites of the national public funding reimbursement decision making agencies [44–53]. The regulatory review and approval procedures were extracted from the assessment reports of novel medicines disclosed by respective medicines regulatory authorities. Detailed data sources see Table S2. Data and information were extracted by two authors in parallel. Any divergence of opinions was discussed with the corresponding author to reach a consensus.

### Statistical analysis

We calculated the number of days between the date that MA was granted and the date that the reimbursement decision of public funding was announced in respective countries. Given the anticipated non-normal distribution of TTR, we conducted the Shapiro-Wilkm tests and Brown-Forsythe tests to check the distributions of TTRs across all countries [54, 55]. Pairwise comparisons between countries were conducted using Kruskal–Wallis *H* tests with Bonferroni corrections [16, 56].

We performed the univariate Cox proportional hazards regression analyses and drew the Kaplan–Meier (KM) curves to describe the distributions of TTRs and the survival rates (non-reimbursed medicines as a proportion to the total number of target medicines) of the target medicines in a pool of five countries and in each country respectively [57]. We then performed the multiple Cox proportional hazards regression by inclusion of country and year dummy variables. Other covariates associated with the characteristics of novel medicines and manufacturers, as well as the review and approval pathways were included in the regression. The Cox Proportional Hazards model is given by the following equation:

$${\text{h}}\left( {t|X} \right) = {\text{h}}_{0} \left( t \right){\text{exp}}(\beta_{{1}} X_{{1}} + \beta_{{2}} X_{{2}} + \cdots + \beta_{{\text{p}}} X_{{\text{p}}} )$$

where h(*t*|*X*) is the hazard function at time *t* given covariates *X*, h_0_(*t*) is the baseline hazard function (the hazard when all covariates are zero), *β*_1,_*β*_2,_…*β*_p_, are the regression coefficients for the covariates, *X*_1_, *X*_2_,…,*X*_p_ are the covariates affecting the survival time [58].

Subgroup analyses were conducted in each country to assess the specific factors associated with the TTR in respective countries with stratified Cox regression analyses. Considered that the annual inclusion of novel medicines through price negotiation in China was institutionalized since 2019, which might increase the number of novel medicines reimbursed by public funding. To ensure consistency of the data collected from China, we performed the sensitivity analyses by removing the data of 2018 to repeat the analyses. This is expected to help mitigate the potential bias brought by the accumulated non-reimbursed novel medicines approved in 2018, when systematic reimbursement of novel medicines by public funding yet started. The threshold for statistical significance was set at a two-tailed *P* < 0.05 for all analyses. All statistical analyses were performed using Stata/MP 17.0 and R version 4.2.2.

## Results

### Characteristics of novel medicines

845 of 1186 novel medicines approved during 2018 and 2023, and 699 of 1000 novel medicines approved during 2019 and 2023 in five countries were publicly funded as of July 1st, 2024. Japan had the highest proportion of publicly funded novel medicines (93.36%, n = 211), followed by France (72.53%, n = 169), the UK (69.30%, n = 158), China (62.00%, n = 186), and Switzerland (60.80%, n = 121). Characteristics of these medicines were presented in Table S3. Detailed descriptions in different subgroups were shown in Table S4.

### Comparison of TTR

**Fig. 1:**
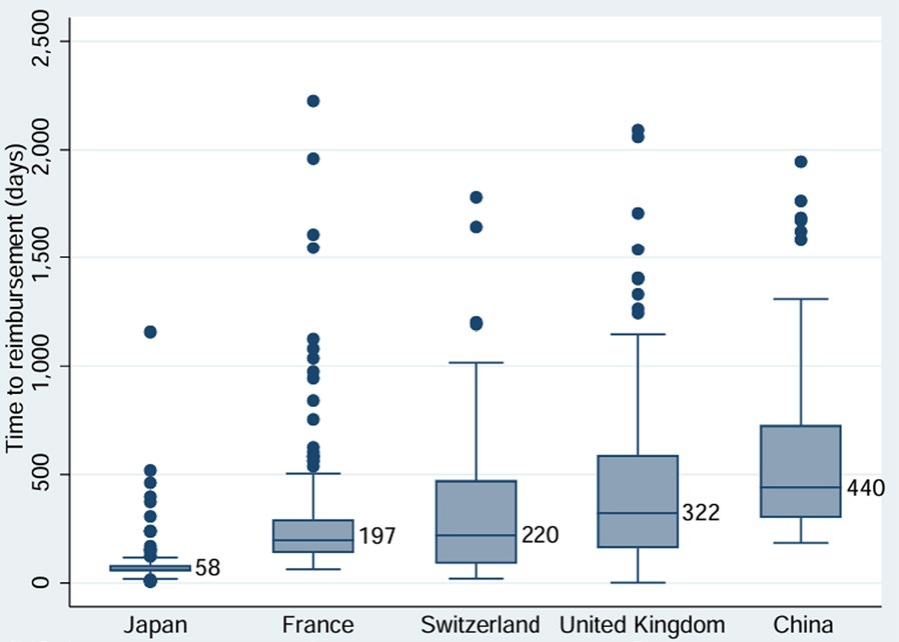
Boxplots of time to reimbursement of publicly funded novel medicine in five Eurasian countries

Figure 1 presented the median TTRs (mTTRs) of novel medicines approved in five countries during 2018 and 2023. The overall mTTR was 211 days (1-2225). Japan had the shortest mTTR of 58 days (8-1156), followed by France with 197 days (63-2225), Switzerland with 220 days (20-1781), and the UK with 322 days (1-2091). China had the longest mTTR of 440 days (185-1945). Detailed descriptions of mTTRs in different subgroups were shown in Table S4.

The pairwise comparisons indicated that the TTR in Japan was shorter than that in all other countries, the TTR of China was longer than that in all other countries (Table S5). Following the Bonferroni corrections, there were still statistically significant differences of TTR between Japan and all other countries (Table S6).

### Cox proportional hazards regression analysis results

**Fig. 2:**
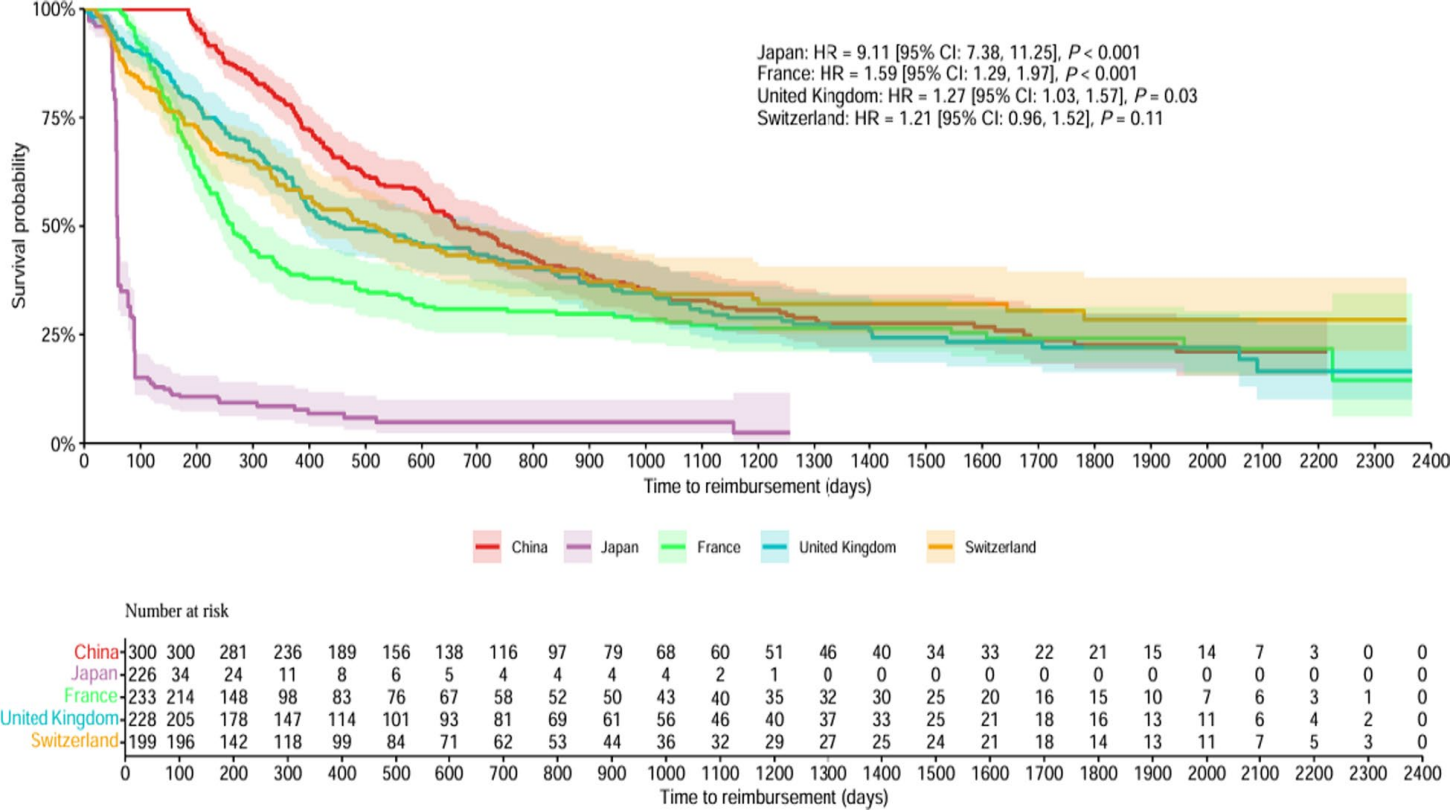
Kaplan–Meyer curves of public funding reimbursement and time to reimbursement by country

**Fig. 3:**
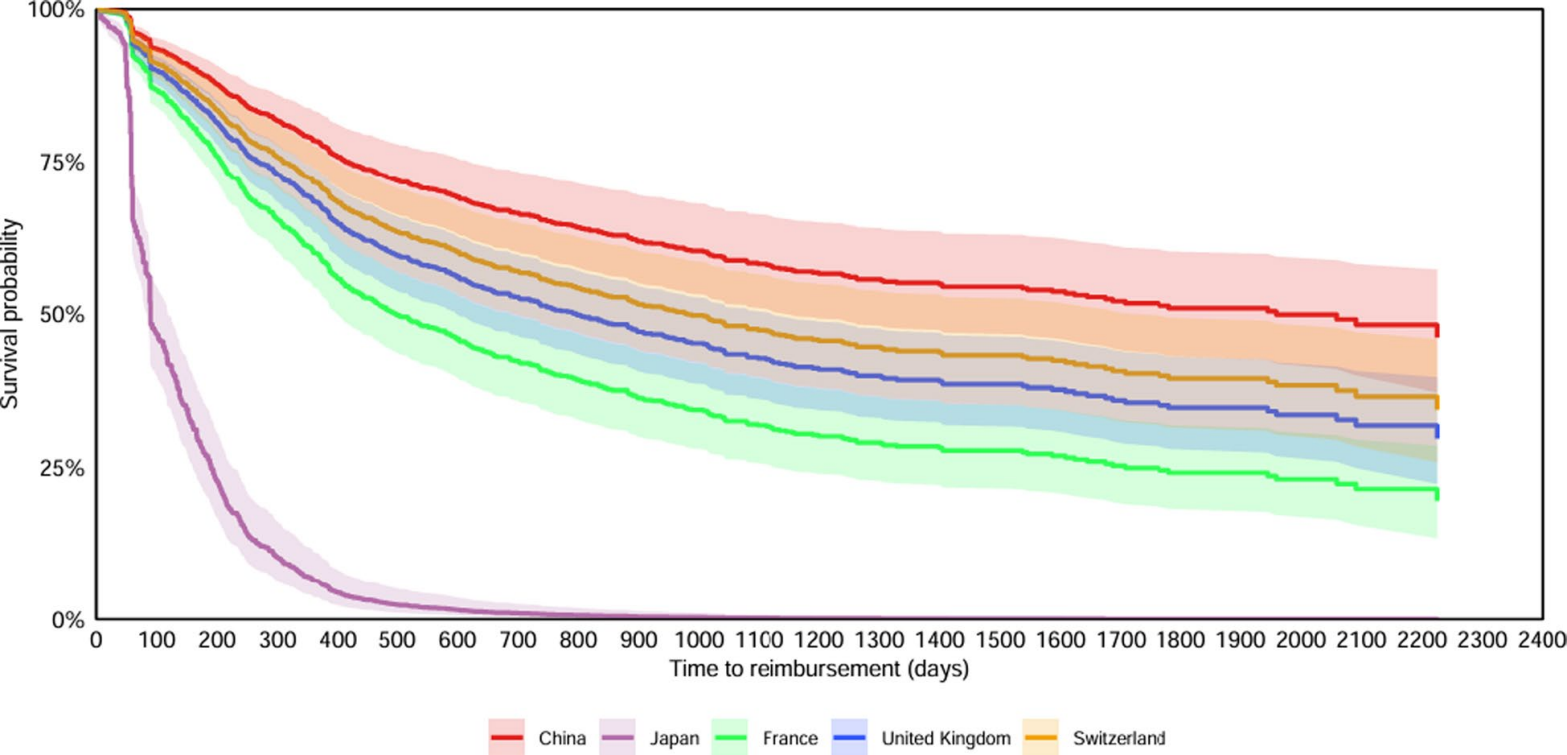
Adjusted Cox proportional hazards regression analysis in five Eurasian countries

**Fig. 4:**
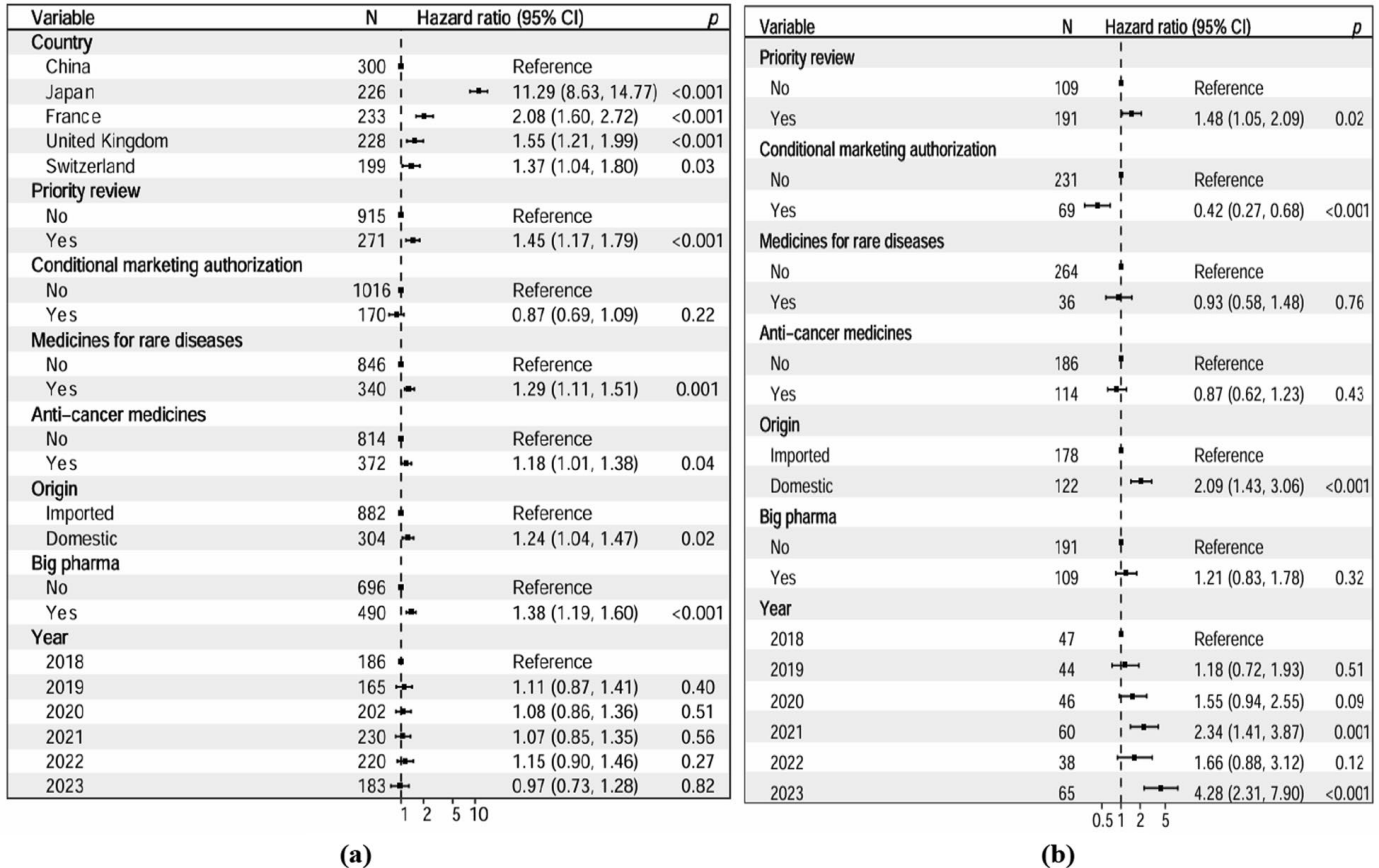
Hazard Ratios for reimbursement of novel medicines by public funding in five Eurasian countries (**a**) and in China (**b**)

The KM curves of public funding reimbursement and TTR by countries were presented in Fig. 2 and in Figure S1–S7 by covariates. By adjusting for all the covariates, the multiple Cox proportional hazards regression showed that novel medicines approved in Japan (Hazard Ratio (HR) = 11.29, [95% CI 8.63, 14.77], *P* < 0.001), France (HR = 2.08, [95% CI 1.60, 2.72], *P* < 0.001), the UK (HR = 1.55, [95% CI 1.21, 1.99], *P* = 0.001), and Switzerland (HR = 1.37, [95% CI 1.04, 1.80], *P* = 0.03) were more likely to be publicly funded than that approved in China. Novel medicines approved through PR procedure (HR = 1.45, [95% CI 1.17, 1.79], *P* = 0.001), novel medicines for RDs (HR = 1.29, [95% CI 1.11, 1.51], *P* = 0.001), novel anti-cancer medicines (HR = 1.18, [95% CI 1.01, 1.38], *P* = 0.04), locally developed novel medicines (HR = 1.24, [95% CI 1.04, 1.47], *P* = 0.02), and novel medicines launched by big pharma (HR = 1.38, [95% CI 1.19, 1.60], *P* < 0.001) were more likely to be publicly funded (Figs. 3, 4, Table S7,S8).

### Subgroup analyses

HR for public funding reimbursement of novel medicines in China indicated that, novel medicines approved through PR procedure (HR = 1.48, 95% CI [1.05, 2.09], *P* = 0.03) and locally developed novel medicines (HR = 2.09, 95% CI [1.43, 3.06], *P* < 0.001) were more likely to be publicly funded, those approved through CMA were less likely to be publicly funded (HR = 0.42, 95% CI [0.27, 0.68], *P* < 0.001). In Japan, novel medicines for RDs were more likely to be publicly funded (HR = 1.52, 95% CI [1.11, 2.08], *P* = 0.01). In France, novel medicines for RDs were more likely to be publicly funded (HR = 1.45, 95% CI [1.04, 2.04], *P* = 0.03) In the UK, novel medicines approved through PR procedure (HR = 2.38, 95% CI [1.19, 4.74], *P* = 0.01), novel anti-cancer medicines (HR = 1.50, 95% CI [1.01, 2.22], *P* = 0.04) were more likely to be publicly funded** (**Figure S8–S11).

### Sensitivity analyses

By removing the data of 2018, the mTTRs of reimbursed novel medicines approved in five countries during 2019 and 2023 remained consistent with the original analysis results**.** Novel medicines approved in China remained less likely to be publicly funded than that approved in Japan, France, and the UK. The results of all the sensitivity analyses were consistent with that of the original analyses (Figure S12, 13 Table S9–12**).**

## Discussion

Japan had the highest proportion of novel medicines reimbursed by public funding and with the fastest rate. The proportion and the rate were the lowest in China. Shorter TTR was found in China after 2019 when annual inclusion of novel medicines in the national basic health insurance through national price negotiation was institutionalized. This narrowed down the gap of TTR between China and the other countries. These were consistent with the findings of Liu and Xia’s studies [2, 3]. Compared with the other countries which already established mature public funding reimbursement of novel medicines, China still has a long way to go. Japan holds four evaluation meetings annually. Novel medicines are publicly funded within 60 to 90 days after obtaining MA [59]. In France, the decision of public funding of novel medicines must be made within 180 days after application [60]. The UK adopts different HTA methods for different novel medicines and includes novel medicines into the NHS irregularly through publication of guidelines. Application for public funding of novel medicines in China is scheduled in every July. The evaluation and price negotiation complete by the end of the same year. Unless a novel medicine is granted with MA in the first half of a year, it must wait until the next year for application [3].

For novel medicines with major public health or therapeutic significance to address the unmet clinical needs, they are reviewed and approved through PR procedure with accelerated MA in all countries. Novel medicines approved through PR procedure in the UK were more likely to be publicly funded, which is related to UK’s efforts to expedite the clinical study, data generation and collection, MA and public funding reimbursement of novel medicines with promising potential through the Early Access to Medicines Scheme (EAMS) and the Innovative Licensing and Access Pathway (ILAP) [61, 62]. Pharmaceutical companies are offered with joint technical guidance by the Medicines and Healthcare Products Regulatory Agency and the NICE during preclinical research or the early stage of clinical trials. This process ensures that clinical study design and data collection meet the technical requirements, and speeds up the MA and public funding reimbursement assessments. EAMS also allows companies to provide free medicines to patients 12–18 months prior to MA, for collection of real-world evidence. This enables MA and public funding reimbursement assessments to be conducted simultaneously. NHS is obligated to pay within 3 months after a positive HTA opinion given by NICE. Additionally, for novel medicines approved through EAMS, NHS needs to pay within 30 days after obtaining a positive HTA opinion [62]. Similar with the EAMS and ILAP approaches adopted in the UK, Japan also provides advanced HTA technical support to innovative pharmaceutical companies at pre-clinical and clinical research stage through “SAKIGAKE” designation, which facilitates appropriate evidence generation and preparedness for HTA [63]. In China, MA and public funding reimbursement assessments are separate procedures with no overlap. Both the national medicines regulatory authority and the national public funding reimbursement decision making agency started to have active communications with the applying companies. However, the latter only interacts with the pharmaceutical companies after obtaining MA. No technical guidance is provided at the clinical research stage. The Chinese national health insurance should adopt more forward-looking proactive approach like that implemented in the UK.

China is the only country that novel medicines approved through CMA were less likely to be publicly funded, which is consistent with the findings of Zhu’s study [22]. This might be associated with the fact that, apart from China, all other countries established the managed entry agreements (MEAs) between the pharmaceutical companies and the payers for expensive novel medicines with clinical and cost uncertainties [64, 65]. The UK implements the MEA and the Patient Access Scheme for high-value novel medicines. A series of payment agreements and real-world clinical evidence collection plans have been developed. NHS and pharmaceutical companies share the responsibility of accelerating patient access to novel medicines, and managing the potential risks arising from the uncertainties related to clinical outcomes and long-term costs [4, 64, 65]. Novel medicines approved through CMA have not yet completed phase III clinical trials, and with clinical and budget impact uncertainties. China might be discreet with such novel medicines to minimize the potential risks related to efficacy, safety, and budget impact. For medicines approved through CMA, the Chinese national health insurance could leverage the international experiences to make payment agreement with pharmaceutical companies based on the real-world evidence. These agreements allow risk-sharing between the public funding agencies and the pharmaceutical companies via accelerated public funding reimbursement of novel medicines that address the urgent clinical needs but with certain uncertainties. The agreement would also be helpful to control the post-market surveillance and the budget impact.

Most RDs are caused by genetic mutations, manifesting at birth or in childhood, imposing a significant burden on patients, families, and society [4, 66]. Novel medicines for RDs were found to be more likely to be publicly funded in Japan and France. This might be associated with the pricing policies for novel medicines in Japan, as pricing is a key part of their public funding reimbursement. Through the SAKIGAKE strategy, Japan accelerates the MA and public funding reimbursement of novel medicines that target incurable conditions and persistent symptoms but lack of treatment options. During the development stage of these medicines, the national medicines regulatory authority and the national public funding reimbursement decision making agency provide collaborate technical supports to pharmaceutical companies, which reduces half of the review time for public funding reimbursement decision making. Furthermore, Japan established earmarked funding for RDs, supported by special allocations such as consumption tax. This type of funding allows higher pricing for novel medicines targeted RDs [67]. In France, clinical benefit assessment and improved clinical benefit assessment results are key considerations for public funding reimbursement of novel medicines. Novel medicines cover unmet clinical needs like RDs are classified as with major clinical benefits and significant improvement of clinical benefits, which are allowed to be priced higher and granted with higher reimbursement rate [60, 66]. Novel medicines for RDs typically address unmet clinical needs with significant improvements in clinical benefits, thus are more likely to be supported by public funding [4, 66, 68]. We did not find similar advantages of novel medicines for RDs in China. Although novel medicines that are urgently needed in China (including the imported ones and those for RDs), are allowed to be used in the designated health institutions of Hainan province and Beijing, and the clinical evidence generated in the real world could be used to support regulatory approval. This is now limited to a small number of pilot regions, and the costs of novel medicines are still mainly borne by the patients.

Novel anti-cancer medicines were found to be more likely to be publicly funded in the UK, driven by the proactive policies to improve patient access. Anti-cancer medicines with insufficient clinical evidence or an incremental cost-effectiveness ratio that exceeds the payment threshold, but with expected therapeutic benefits, could be put into clinical use and paid by the Cancer Drugs Fund (evolved into the Innovative Medicines Fund) [69]. This allows certain anti-cancer medicines with clinical and cost uncertainties temporarily funded by alternative source of funding. During this period, pharmaceutical companies can collect real-world clinical data for further NICE assessment [70]. Pharmaceutical companies are required to share the additional costs proportionally when expenditures exceed the fixed budget, with no upper limit [69, 70]. France enacted the Social Security Financing Law in 2021 to allow unapproved innovative medicines for urgent clinical needs or serious diseases to be reimbursed through Temporary Use Authorizations [71]. These proactive strategies that grant temporary reimbursement of innovative medicines lead to faster public coverage of new treatments for cancer patients and other serious conditions. With the increasing number and the high prices of anti-cancer novel medicines, China also needs alternative funding source to pay for the real-world use of anti-cancer novel medicines before public funding reimbursement.

Locally developed novel medicines were found to be more likely to be publicly funded in China. This might be attributed to the strong government support to local innovation. The National Major Novel Medicines Development Program, the National Major Science and Technology Project, and the National Science and Technology Progress Awards are important advantages for novel medicines considered to be publicly funded. Moreover, local developers might face fewer constraints from global pricing strategies compared to the multinational pharmaceutical companies, which enables them more flexible in price negotiation and public funding reimbursement. Thus, in the scenarios where there are no significant differences in efficacy and safety, locally developed novel medicines often have the advantage to be publicly funded.

This study has several limitations. Firstly, public funding reimbursement and TTR of novel medicines were the key outcome variable to measure patient access. And TTR was calculated based on the date of obtaining MA and public funding reimbursement which were extracted from the official websites of the national medicines regulatory authorities and national public funding reimbursement decision making agencies. However, being publicly funded does not guarantee that patients can get access. Appropriate procurement, distribution and prescription, and pharmaceutical benefit package design all affect actual patient access. These might vary across different settings and healthcare systems. Secondly, this study regarded that the PR procedure, CMA, and RDs were the potential attributable factors to address the public health significances and the unmet clinical needs. The study did not quantify the novelty or clinical importance of the target medicines. Pricing factor was also not considered. Furthermore, the market size, capacity of public funding systems, and the political, economic and social complexities of different countries may also affect the analysis results. Although the country-specific dummy variables could help to control these factors to some extents, it still warrants further in-depth research.

## Conclusions

Compared to other global pharmaceutical innovation leaders, China still needs to make further efforts in strengthening public funding reimbursement of novel medicines. A forward-thinking strategy for HTA that provides advanced technical support to pharmaceutical companies in conjunction with the medicines regulatory authority is critical for reducing TTR and accelerating patient access. Risk-sharing mechanism for novel medicines with clinical and cost uncertainties, temporary reimbursement with alternative sources of funding to support real-world evidence collection could be the strategies to help strike a balance between timely patient access to innovative treatments and maintaining effective risk management.

## Supplementary Information

Additional file1 (DOCX 2239 kb)

## Data Availability

Our study is based on published data. Therefore, there are no primary data to be shared. The data supporting the findings of our study are available from the Supplementary material. The statistical plan and code for analyses will be available upon request from the corresponding author for non-commercial purposes.

## Abbreviations

CMA: Conditional market authorization
EAMS: Early Access to Medicines Scheme
HICs: High-income countries
HR: Hazard ratio
HTA: Health technology assessment
ILAP: Innovative Licensing and Access Pathway
KM: Kaplan–Meier
MA: Market authorization
MEA: Managed entry agreements
mTTR: Median TTR
NHS: National Health Service
NICE: National Institute for Health and Care Excellence
PR: Priority review
RDs: Rare diseases
TTR: Time to reimbursement
UK: United Kingdom
US: United States
