# Supplementary material for: Survival analysis of time to reimbursement of novel medicines in five Eurasian countries
Source: Glob Health Res Policy. 2025 Oct 27;10:52. doi: 10.1186/s41256-025-00457-3 (PMC12557994; doi:10.1186/s41256-025-00457-3)
Supplement: Supplementary file 1 — Additional file1 (DOCX 2239 kb) [file 41256_2025_457_MOESM1_ESM.docx]

**Survival analysis of time to reimbursement of novel medicines in five Eurasian countries**

Supplementary material

Directory

[Table S1: Definitions of novel medicines 1](#_Toc13252)

[Table S2: Data source 2](#_Toc29873)

[Table S4: Time to reimbursement in different settings and different sub-groups 5](#_Toc26242)

[Table S5: Kruskal-Wallis H test for difference in TTR between countries 8](#_Toc1913)

[Table S6: Kruskal-Wallis H test with Bonferroni correction for difference in TTR between countries 8](#_Toc17605)

[Figure S1: Kaplan–Meyer curves of public funding reimbursement and time to reimbursement (by priority review) 9](#_Toc25083)

[Figure S2: Kaplan–Meyer curves of public funding reimbursement and time to reimbursement (by conditional marketing authorization) 10](#_Toc10743)

[Figure S3: Kaplan–Meyer curves of public funding reimbursement and time to reimbursement (by medicines for rare diseases) 11](#_Toc22102)

[Figure S4: Kaplan–Meyer curves of public funding reimbursement and time to reimbursement (by anti-cancer medicines) 12](#_Toc23216)

[Figure S5: Kaplan–Meyer curves of public funding reimbursement and time to reimbursement (by origin) 13](#_Toc11757)

[Figure S6: Kaplan–Meyer curves of public funding reimbursement and time to reimbursement (by big pharma) 14](#_Toc23882)

[Figure S7: Kaplan–Meyer curves of public funding reimbursement and time to reimbursement (by year) 15](#_Toc13867)

[Table S7: Univariate Cox proportional hazard regression analysis in different countries 16](#_Toc4112)

[Table S8: Multiple Cox proportional hazard regression analysis in different countries 20](#_Toc18549)

[Figure S8: Hazard Ratios for public funding reimbursement of novel medicines in Japan 21](#_Toc12792)

[Figure S9: Hazard Ratios for public funding reimbursement of novel medicines in France 22](#_Toc20103)

[Figure S10: Hazard Ratios for public funding reimbursement of novel medicines in United Kingdom 23](#_Toc31235)

[Figure S11: Hazard Ratios for public funding reimbursement of novel medicines in Switzerland 24](#_Toc3787)

[Figure S12: Sensitivity analysis of boxplots of time to reimbursement for the reimbursed novel medicines in five countries 25](#_Toc31409)

[Figure S13: Sensitivity analysis of Kaplan-Meyer curve of time to reimbursement in five countries 26](#_Toc10355)

[Table S9: Sensitivity analysis of Kruskal-Wallis H test for difference in TTR between the countries 27](#_Toc14909)

[Table S10: Sensitivity analysis of Kruskal-Wallis H test with Bonferroni correction for difference in TTR between the countries 27](#_Toc9566)

[Table S11: Sensitivity analysis of univariate Cox proportional hazard regression analysis in all countries and China 28](#_Toc28017)

[Table S12: Sensitivity analysis of multiple Cox proportional hazard regression analysis in all countries and China 30](#_Toc30725)

[Table S13: Cross-country comparisons of mechanisms for reimbursement decision on novel medicines 31](#_Toc29263)

[Appendix: The list of big pharma (sort by alphabet) 32](#_Toc11790)

[Reference 33](#_Toc20717)

Table S1: Definitions of novel medicines

| Settings | Definitions of novel medicines |
| --- | --- |
| All countries | In this study, novel medicines are defined as medicines or combinations containing new active ingredients, including chemical and biological products. This definition excludes new indications, new dosage forms, new administration routes, and new target population. It also excludes generics and biosimilars, vaccines, allergenic products, blood and blood products, plasma derivatives, cell therapy and gene therapy, as well as cosmetics and over-the-counter medicines that are usually not publicly funded.[[1](#_ENREF_1)] |
| **China** | Novel medicines are categorized into four types: traditional Chinese medicine, chemical novel medicines, novel vaccines, and novel biological products. Among these, chemical novel medicines refer to compounds that have not yet been approved either domestically or internationally, containing new and structurally defined compounds with pharmacological effects and clinical value, excluding modified new medicines; novel vaccines refer to vaccines that have not yet been approved either domestically or internationally; novel biological products refer to therapeutic biological products that have not yet been approved at home or abroad.[[2](#_ENREF_2)] In this study, novel medicines are defined as medicines or combinations containing new active ingredients, including chemical and biological products. Chemical medicines include class 1 products (novel medicines not yet approved domestically or internationally) and Class 5.1 products (foreign-approved medicines). Biological products are domestic or imported biologics that are first approved by the National Medical Products Administration. |
| **Japan** | Novel medicines refer to medicines containing new active ingredients, new combinations, new administration routes, new therapeutic effects, new indications, as well as new formulations or new dosages.[[3](#_ENREF_3)] They differ from medicines that have already been approved in terms of active ingredients, dosage form, administration routes, dose, efficacy, and effects significantly. |
| **France** | Medicines that contain new active substance or combination of new active substances that have not been authorized before.[[4](#_ENREF_4)] |
| **United Kingdom** | UK official agency does not provide a specific definition for novel medicines. However, the academic community defines novel medicines as entirely or partially new active substances or biological entities, or combinations of these entities, that combat diseases, alleviate symptoms, or prevent illnesses, and improve patient management and outcomes. It includes new indications, new technologies and manufacturing processes, new formulations, new combinations, and new delivery systems.[[5](#_ENREF_5), [6](#_ENREF_6)] |
| **Switzerland** | The novel medicine is authorized by the Swiss Agency for Therapeutic Products as the first containing a specific new active substance, including all dosage forms authorized simultaneously or subsequently as novel medicine.[[7](#_ENREF_7)] |

Table S2: **Data source**

|  | **China** | **Japan** | **France** | **United Kingdom** | **Switzerland** |
| --- | --- | --- | --- | --- | --- |
| **Market authorization** | "Annual Review Report" published by the National Medical Products Administration.[[8-13](#_ENREF_8)]  Cross-referenced with data of China from the "Global Approved New Drugs" section of the Pharmacodia database.[[14](#_ENREF_14)]  Cross-referenced with the "China Approved Drug System" in the Yaozhi database.[[15](#_ENREF_15)]  Cross-referenced with the "Global New Drug Database" in the Insight database.[[16](#_ENREF_16)] | Filtered the "new active ingredients" from the annual "List of Approved Products" published by the Pharmaceuticals and Medical Devices Agency (PMDA).[[17](#_ENREF_17)]  Cross-referenced with data of Japan from the "Global Approved New Drugs" section of the Pharmacodia database.[[14](#_ENREF_14)]  Cross-referenced with the "Japan Approved Drug System" in the Yaozhi database.[[18](#_ENREF_18)]  Cross-referenced with the "Global New Drug Database" in the Insight database.[[16](#_ENREF_16)] | The list of "Approved medicines" published by the European Medicines Agency (EMA).[[19](#_ENREF_19)]  Cross-referenced with data of France from the "Global Approved New Drugs" section of the Pharmacodia database.[[14](#_ENREF_14)]  Cross-referenced with the "New Active Substances approved by six major authorities" published by the Center for Innovation in Regulatory Science (CIRS) database.[[20-24](#_ENREF_20)]  Cross-referenced with the "EMA Approved Drug System" in the Yaozhi database.[[25](#_ENREF_25)]  Cross-referenced with the "Global New Drug Database" in the Insight database.[[16](#_ENREF_16)] | The list of “Approved Medicines” in the years between 2018 and 2023, published by the Medicines and Healthcare products Regulatory Agency (MHRA).[[26](#_ENREF_26)]  The data prior to Brexit was cross-referenced with UK data from the "Global Approved New Drugs" section of the Pharmacodia database.[[14](#_ENREF_14)]  The data prior to Brexit was cross-referenced with the "New Active Substances approved by six major authorities" published by the CIRS database.[[20-24](#_ENREF_20)]  Cross-referenced with the "EMA Approved Drug System" prior to Brexit,[[25](#_ENREF_25)] and the "UK Approved Drug System" after Brexit[[27](#_ENREF_27)] in the Yaozhi database.  Cross-referenced with the "Global New Drug Database" in the Insight database for the years 2018 to 2023.[[16](#_ENREF_16)] | The List of “Authorised medicinal products" published by the Swiss Agency for Therapeutic Products (Swissmedic).[[28](#_ENREF_28)]  Search for "human medicinal products with a new active substance" in the "Swissmedic Annual Report" published by Swissmedic.[[29-34](#_ENREF_29)]  Cross-referenced with the "Global Approved Drug Screening System" in the Yaozhi database.[[35](#_ENREF_35)]  Cross-referenced with the "New Active Substances approved by six major authorities" published by the CIRS database.[[20-24](#_ENREF_20)]  Cross-referenced with the "Global New Drug Database" in the Insight database[[16](#_ENREF_16)] |
| **Public funding reimbursement** | The annual national basic health insurance reimbursement list of medicines, published annually by the National Healthcare Security Administration to obtain the public funding reimbursement date.[[36-41](#_ENREF_36)] | The meeting records of the Central Social Insurance Medical Council, published by the Ministry of Health, Labour and Welfare to obtain the public funding reimbursement date.[[42](#_ENREF_42)] | Search the names of novel medicines published on the official website of the Haute Autorité de Santé in the section of "All publications-Recommendations, medications, procedures, devices, etc." to obtain the public funding reimbursement date.[[43](#_ENREF_43)] | Search the names of novel medicines published on the official website of the National Institute for Health and Care Excellence (NICE) in the section of "NICE guidance" to obtain the corresponding guideline documents. The publication date of the guideline was regarded as the public funding reimbursement date.[[44](#_ENREF_44)] | Search fthe names of novel medicines in the Specialty List published by the Federal Office of Public Health to obtain the public funding reimbursement date.[[45](#_ENREF_45)] |
| **Priority review** | Description of "priority review procedure" extracted from the first Technical Review Report of the target novel medicines.[[46](#_ENREF_46)]  Cross-referenced with the "Annual Review Reports" regarding the expedited approval pathways.[[8-13](#_ENREF_8)] | Filtered the "priority review" and "SAKIGAKE designation" from the annual list of “Approved Products" published by the PMDA.[[17](#_ENREF_17)]  Cross-referenced with the list of “Products subject to SAKIGAKE designation System and the SAKIGAKE designation System" published by the PMDA.[[47](#_ENREF_47)] | Filtered "accelerated assessment" from the list of "Approved medicines" published by the EMA.[[19](#_ENREF_19)]  Cross-referenced with the "Assessment report" by searching “accelerated assessment”.[[48](#_ENREF_48)]  Cross-referenced with the "expedited review" section for France from the "New Active Substances approved by six major authorities" published by the CIRS database.[[20-24](#_ENREF_20)] | "Early access to medicines scheme: expired scientific opinions" published by the MHRA.[[49](#_ENREF_49)]  The data prior to Brexit was cross-referenced with the "Accelerated assessment" in the list of "Approved medicines" published by the EMA.[[19](#_ENREF_19)]  The data prior to Brexit was cross-referenced with the "expedited review" section for UK from the "New Active Substances approved by six major authorities", published by the CIRS database.[[20-24](#_ENREF_20)] | Cross-referenced with the "expedited review" section for Switzerland from the "New Active Substances approved by six major authorities", published by the CIRS database.[[20-24](#_ENREF_20)] |
| **Conditional market authorization** | Description of "conditional market authorization" in the first technical review report of the target novel medicines.[[46](#_ENREF_46)]  Cross-referenced with the "Annual Review Reports" regarding the expedited approval pathways.[[8-13](#_ENREF_8)] | Filtered "conditional early approval" from the annual list of “Approved Products" published by the PMDA.[[17](#_ENREF_17)]  Cross-referenced with "the conditional early approval system for pharmaceuticals" published by the PMDA.[[50](#_ENREF_50)] | Filterteed "conditional approval" in the list of "Approved medicines" published by the EMA.[[19](#_ENREF_19)]  Cross-referenced with the "Assessment report" by searching “conditional approval”.[[48](#_ENREF_48)]  Cross-referenced with the "conditional approval" section for France from the "New Active Substances approved by six major authorities", published by the CIRS database.[[20-24](#_ENREF_20)] | The data prior to Brexit was cross-referenced with the "conditional approval" in the list of "Approved medicines" published by the EMA.[[19](#_ENREF_19)]  The data prior to Brexit was cross-referenced with the "conditional approval" section for UK from the "New Active Substances approved by six major authorities" published by the CIRS database.[[20-24](#_ENREF_20)]  After Brexit, search for "conditional marketing authorisation" in the "Public Assessment Report" published by MHRA.[[51](#_ENREF_51)] | The list of "Temporarily authorised human medicines" published by the Swissmedic.[[52](#_ENREF_52)]  Cross-referenced with the "Temporary authorisation" section in the "Swiss Public Assessment Report" (SwissPAR) published by the Swissmedic.[[53](#_ENREF_53)]  Cross-referenced with the "conditional approval" section for Switzerland from the "New Active Substances approved by six major authorities" published by the CIRS database.[[20-24](#_ENREF_20)] |
| **Medicines for rare diseases** | Medicines for rare diseases were judged based on the first and second lists of rare diseases published by the National Health Commission.[[54](#_ENREF_54), [55](#_ENREF_55)]  Description of "rare disease" in the first Technical Review Report of the target novel medicines.[[46](#_ENREF_46)] | Filtered "orphan drug" from the annual list of “Approved Products" published by the PMDA.[[17](#_ENREF_17)]  Cross-referenced with the "List of Designated Drugs for Orphan Diseases" published by the PMDA.[[56](#_ENREF_56)] | Filtered "orphan medicine" from the list of "Approved medicines" published by the EMA.[[19](#_ENREF_19)]  Cross-referenced with the "Assessment report" by searching “orphan drug”.[[48](#_ENREF_48)]  Cross-referenced with the list of “Opinions on Orphan Medicinal Product Designation" published by the EMA.[[57](#_ENREF_57), [58](#_ENREF_58)]  Cross-referenced with the "orphan" section for France from the "New Active Substances approved by six major authorities" published by the CIRS database.[[20-24](#_ENREF_20)] | The "Orphan register" website.[[59](#_ENREF_59)]  The data prior to Brexit was cross-referenced with the "orphan medicine" in the list of "Approved medicines" published by the EMA.[[19](#_ENREF_19)]  The data prior to Brexit was cross-referenced with the "orphan" section for UK from the "New Active Substances approved by six major authorities" published by the CIRS database.[[20-24](#_ENREF_20)]  After Brexit, searched "orphan drug" in the "Public Assessment Report" published by the MHRA.[[51](#_ENREF_51)] | The list of "Important medicinal products for rare diseases (orphan drugs in the case of human medicines)" published by the Swissmedic.[[60](#_ENREF_60)]  Cross-referenced "orphan drug status" in the SwissPAR published by the Swissmedic.[[53](#_ENREF_53)]  Cross-referenced with the "orphan" section for Switzerland from the "New Active Substances approved by six major authorities" published by the CIRS database.[[20-24](#_ENREF_20)] |

Note: PMDA = Pharmaceuticals and Medical Devices Agency; EMA = European Medicines Agency; CIRS = Center for Innovation in Regulatory Science; MHRA = Medicines and Healthcare products Regulatory Agency; NHSA = National Healthcare Security Administration; NICE = National Institute for Health and Care Excellence; PAR = Public Assessment Report; SwissPAR = Swiss Public Assessment Report; Expedited review refers to the accelerated assessment in EMA and the fast-track procedure in Switzerland.

**Table S3: Summary of characteristics of target medicines**

|  | **No. of novel medicines covered by public funding/No. of novel medicines obtained market authorization (%)** | | |
| --- | --- | --- | --- |
|  | **2018-2023** | | **2019-2023** |
| All countries | 845/1186 (71.25%) | 699/1000 (69.90%) | |
| Country |  |  | |
| China | 186/300 (62.00%) | 153/253 (60.47%) | |
| Japan | 211/226 (93.36%) | 176/191 (92.15%) | |
| France | 169/233 (72.53%) | 137/195 (70.26%) | |
| United Kingdom | 158/228 (69.30%) | 129/190 (67.89%) | |
| Switzerland | 121/199 (60.80%) | 104/171 (60.82%) | |
| Year |  |  | |
| 2018 | 146/186 (78.49%) |  | |
| 2019 | 131/165 (79.39%) | 131/165 (79.39%) | |
| 2020 | 155/202 (76.73%) | 155/202 (76.73%) | |
| 2021 | 181/230 (78.70%) | 181/230 (78.70%) | |
| 2022 | 147/220 (66.82%) | 147/220 (66.82%) | |
| 2023 | 85/183 (46.45%) | 85/183 (46.45%) | |
| Priority review |  |  | |
| No | 648/915 (70.82%) | 541/779 (69.45%) | |
| Yes | 197/271 (72.69%) | 158/221 (71.49%) | |
| Conditional marketing authorization | |  | |
| No | 738/1016 (72.64%) | 594/835 (71.14%) | |
| Yes | 107/170 (62.94%) | 105/165 (63.64%) | |
| Medicines for rare diseases | |  | |
| No | 585/846 (69.15%) | 486/715 (67.97%) | |
| Yes | 260/340 (76.47%) | 213/285 (74.74%) | |
| Anti-cancer medicines | |  | |
| No | 576/814 (70.76%) | 473/679 (69.66%) | |
| Yes | 269/372 (72.31%) | 226/321 (70.40%) | |
| Origin |  |  | |
| Imported | 622/882 (70.52%) | 509/735 (69.25%) | |
| Domestic | 223/304 (73.36%) | 190/265 (71.70%) | |
| Big pharma |  |  | |
| No | 472/696 (67.82%) | 397/596 (66.61%) | |
| Yes | 373/490 (76.12%) | 302/404 (74.75%) | |

Table S4: **Time to reimbursement in different settings and different sub-groups**

| **Settings and sub-groups** | | | **No. of novel medicines reimbursed by public funding by July1,2024 /No. of novel medicines obtained MA (%)** | **Median TTR (min-max) (days)** |
| --- | --- | --- | --- | --- |
| **All countries** | All medicines | | 845/1186 (71.25) | 221 (1-2225) |
|  | Country | China | 186/300 (62.00) | 441 (185-1945) |
|  |  | Japan | 211/226 (93.36) | 58 (8-1156) |
|  |  | France | 169/233 (72.53) | 197 (63-2225) |
|  |  | United Kingdom | 158/228 (69.30) | 322 (1-2091) |
|  |  | Switzerland | 121/199 (60.80) | 220 (20-1781) |
|  | Year | 2018 | 146/186 (78.49) | 261 (8-2225) |
|  |  | 2019 | 131/165 (79.39) | 324 (8-1781) |
|  |  | 2020 | 155/202 (76.73) | 272 (8-1405) |
|  |  | 2021 | 181/230 (78.70) | 211 (7-1156) |
|  |  | 2022 | 147/220 (66.82) | 134 (8-789) |
|  |  | 2023 | 85/183 (46.45) | 185 (1-358) |
|  | Priority review | No | 648/915 (70.82) | 167 (8-2225) |
|  |  | Yes | 197/271 (72.69) | 384 (1-1945) |
|  | Conditional marketing authorization | No | 738/1016 (72.64) | 196 (7-2225) |
|  |  | Yes | 107/170 (62.94) | 294 (1-1007) |
|  | Medicines for rare diseases | No | 585/846 (69.15) | 216 (7-2225) |
|  |  | Yes | 260/340 (76.47) | 197 (1-2091) |
|  | Anti-cancer medicines | No | 576/814 (70.76) | 199 (7-2225) |
|  |  | Yes | 269/372 (72.31) | 236 (1-1707) |
|  | Origin | Imported | 622/882 (70.52) | 225 (1-2225) |
|  |  | Domestic | 223/304 (73.36) | 188 (19-1404) |
|  | Big pharma | No | 472/696 (67.82) | 234 (7-2091) |
|  |  | Yes | 373/490 (76.12) | 194 (1-2225) |
| **China** | All medicines | | 186/300 (62.00) | 441 (185-1945) |
|  | Year | 2018 | 33/47 (70.21) | 660 (215-1945) |
|  |  | 2019 | 33/44 (75.00) | 737 (372-1764) |
|  |  | 2020 | 33/46 (71.74) | 386 (215-1135) |
|  |  | 2021 | 45/60 (75.00) | 425 (190-1007) |
|  |  | 2022 | 18/38 (47.37) | 392 (246-656) |
|  |  | 2023 | 24/65 (36.92) | 234 (185-356) |
|  | Priority review | No | 61/109 (55.96) | 425 (185-1764) |
|  |  | Yes | 125/191 (65.45) | 462 (185-1945) |
|  | Conditional marketing authorization | No | 154/231 (66.67) | 450 (185-1945) |
|  |  | Yes | 32/69 (46.38) | 410 (199-1007) |
|  | Medicines for rare diseases | No | 161/264 (60.98) | 442 (185-1764) |
|  |  | Yes | 25/36 (69.44) | 436 (185-1945) |
|  | Anti-cancer medicines | No | 122/186 (65.59) | 442 (185-1945) |
|  |  | Yes | 64/114 (56.14) | 435 (188-1674) |
|  | Origin | Imported | 103/178 (57.87) | 593 (214-1945) |
|  |  | Domestic | 83/122 (68.03) | 372 (185-1303) |
|  | Big pharma | No | 120/191 (62.83) | 406 (185-1945) |
|  |  | Yes | 66/109 (60.55) | 577 (214-1764) |
| **Japan** | All medicines | | 211/226 (93.36) | 58 (8-1156) |
|  | Year | 2018 | 35/35 (100.00) | 60 (8-123) |
|  |  | 2019 | 37/37 (100.00) | 57 (8-519) |
|  |  | 2020 | 37/37 (100.00) | 58 (8-462) |
|  |  | 2021 | 44/47 (93.62) | 58 (50-1156) |
|  |  | 2022 | 45/46 (97.83) | 59 (8-398) |
|  |  | 2023 | 13/24 (54.17) | 58 (8-156) |
|  | Priority review | No | 194/209 (92.82) | 59 (8-1156) |
|  |  | Yes | 17/17 (100.00) | 56 (19-89) |
|  | Conditional marketing authorization | No | 208/223 (93.27) | 58 (8-1156) |
|  |  | Yes | 3/3 (100.00) | 56 (54-60) |
|  | Medicines for rare diseases | No | 140/152 (92.11) | 59 (19-1156) |
|  |  | Yes | 71/74 (95.95) | 58 (8-240) |
|  | Anti-cancer medicines | No | 157/169 (92.90) | 58 (8-1156) |
|  |  | Yes | 54/57 (94.74) | 58 (49-154) |
|  | Origin | Imported | 113/123 (91.87) | 58 (8-462) |
|  |  | Domestic | 98/103 (95.15) | 58 (19-1156) |
|  | Big pharma | No | 123/132 (93.18) | 58 (8-1156) |
|  |  | Yes | 88/94 (93.62) | 59 (8-462) |
| **France** | All medicines | | 169/233 (72.53) | 197 (63-2225) |
|  | Year | 2018 | 32/38 (84.21) | 261 (76-2225) |
|  |  | 2019 | 20/29 (68.97) | 293 (128-1608) |
|  |  | 2020 | 25/38 (65.79) | 176 (75-944) |
|  |  | 2021 | 35/46 (76.09) | 166 (63-1125) |
|  |  | 2022 | 35/52 (67.31) | 177 (68-582) |
|  |  | 2023 | 22/30 (73.33) | 186 (93-336) |
|  | Priority review | No | 162/226 (71.68) | 200 (63-2225) |
|  |  | Yes | 7/7 (100.00) | 195 (103-975) |
|  | Conditional marketing authorization | No | 143/199 (71.86) | 205 (63-2225) |
|  |  | Yes | 26/34 (76.47) | 190 (68-754) |
|  | Medicines for rare diseases | No | 107/157 (68.15) | 195 (63-2225) |
|  |  | Yes | 62/76 (81.58) | 206 (65-1036) |
|  | Anti-cancer medicines | No | 115/160 (71.88) | 196 (63-2225) |
|  |  | Yes | 54/73 (73.97) | 200 (77-754) |
|  | Origin | Imported | 162/223 (72.65) | 197 (63-2225) |
|  |  | Domestic | 7/10 (70.00) | 219 (139-288) |
|  | Big pharma | No | 93/134 (69.40) | 237 (65-1959) |
|  |  | Yes | 76/99 (76.77) | 173 (63-2225) |
| **United Kingdom** | All medicines | | 158/228 (69.30) | 322 (1-2091) |
|  | Year | 2018 | 29/38 (76.32) | 539 (86-2091) |
|  |  | 2019 | 22/29 (75.86) | 475 (117-1707) |
|  |  | 2020 | 29/43 (67.44) | 384 (12-1405) |
|  |  | 2021 | 35/44 (79.55) | 269 (7-1146) |
|  |  | 2022 | 28/44 (63.64) | 224 (13-789) |
|  |  | 2023 | 15/30 (50.00) | 127 (1-358) |
|  | Priority review | No | 124/188 (65.96) | 322 (12-2091) |
|  |  | Yes | 34/40 (85.00) | 324 (1-1405) |
|  | Conditional marketing authorization | No | 134/199 (67.34) | 326 (7-2091) |
|  |  | Yes | 24/29 (82.76) | 260 (1-833) |
|  | Medicines for rare diseases | No | 109/158 (68.99) | 324 (7-1707) |
|  |  | Yes | 49/70 (70.00) | 320 (1-2091) |
|  | Anti-cancer medicines | No | 102/160 (63.75) | 371 (7-2091) |
|  |  | Yes | 56/68 (82.35) | 247 (1-1707) |
|  | Origin | Imported | 145/206 (70.39) | 329 (1-2091) |
|  |  | Domestic | 13/22 (59.09) | 454 (82-1404) |
|  | Big pharma | No | 87/138 (63.04) | 390 (7-2091) |
|  |  | Yes | 71/90 (78.89) | 251 (1-1707) |
| **Switzerland** | All medicines | | 121/199 (60.8) | 220 (20-1781) |
|  | Year | 2018 | 17/28 (60.71) | 168 (49-1644) |
|  |  | 2019 | 19/26 (73.08) | 101 (30-1781) |
|  |  | 2020 | 31/38 (81.58) | 518 (21-1200) |
|  |  | 2021 | 22/33 (66.67) | 386 (39-702) |
|  |  | 2022 | 21/40 (52.50) | 135 (24-534) |
|  |  | 2023 | 11/34 (32.35) | 178 (20-343) |
|  | Priority review | No | 107/183 (58.47) | 206 (20-1781) |
|  |  | Yes | 14/16 (87.50) | 345 (40-1200) |
|  | Conditional marketing authorization | No | 99/164 (60.37) | 203 (21-1781) |
|  |  | Yes | 22/35 (62.86) | 363 (20-888) |
|  | Medicines for rare diseases | No | 68/115 (59.13) | 156 (21-1016) |
|  |  | Yes | 53/84 (63.10) | 302 (20-1781) |
|  | Anti-cancer medicines | No | 80/139 (57.55) | 187 (21-1781) |
|  |  | Yes | 41/60 (68.33) | 307 (20-1200) |
|  | Origin | Imported | 99/152 (65.13) | 220 (21-1781) |
|  |  | Domestic | 22/47 (46.81) | 223 (20-1190) |
|  | Big pharma | No | 49/101 (48.51) | 225 (24-1781) |
|  |  | Yes | 72/98 (73.47) | 216 (20-1200) |

Table S5: Kruskal-Wallis H test for difference in TTR between countries

|  | **China** | **Japan** | **France** | **United Kingdom** | **Switzerland** |
| --- | --- | --- | --- | --- | --- |
| **China** |  |  |  |  |  |
| **Japan** | **<0.000001** |  |  |  |  |
| **France** | **<0.000001** | **<0.000001** |  |  |  |
| **United Kingdom** | **0.001** | **<0.000001** | **0.006** |  |  |
| **Switzerland** | **0.000005** | **<0.000001** | 0.15 | 0.08 |  |

**Notes:** Bold means statistically significant.

Table S6: Kruskal-Wallis H test with B**onferroni correction** for difference in TTR between countries

|  | **China** | **Japan** | **France** | **United Kingdom** | **Switzerland** |
| --- | --- | --- | --- | --- | --- |
| **China** |  |  |  |  |  |
| **Japan** | **<0.001** |  |  |  |  |
| **France** | **0.001** | **<0.001** |  |  |  |
| **United Kingdom** | >0.99 | **<0.001** | 0.15 |  |  |
| **Switzerland** | 0.16 | **<0.001** | >0.99 | >0.99 |  |

**Notes:** Bold means statistically significant. Given the number of statistical tests performed within this study we used a Bonferroni correction. For this correction we corrected for individual comparisons between countries. The correction used for the paired differences between the countries was the number of individual paired comparisons (n = 10).


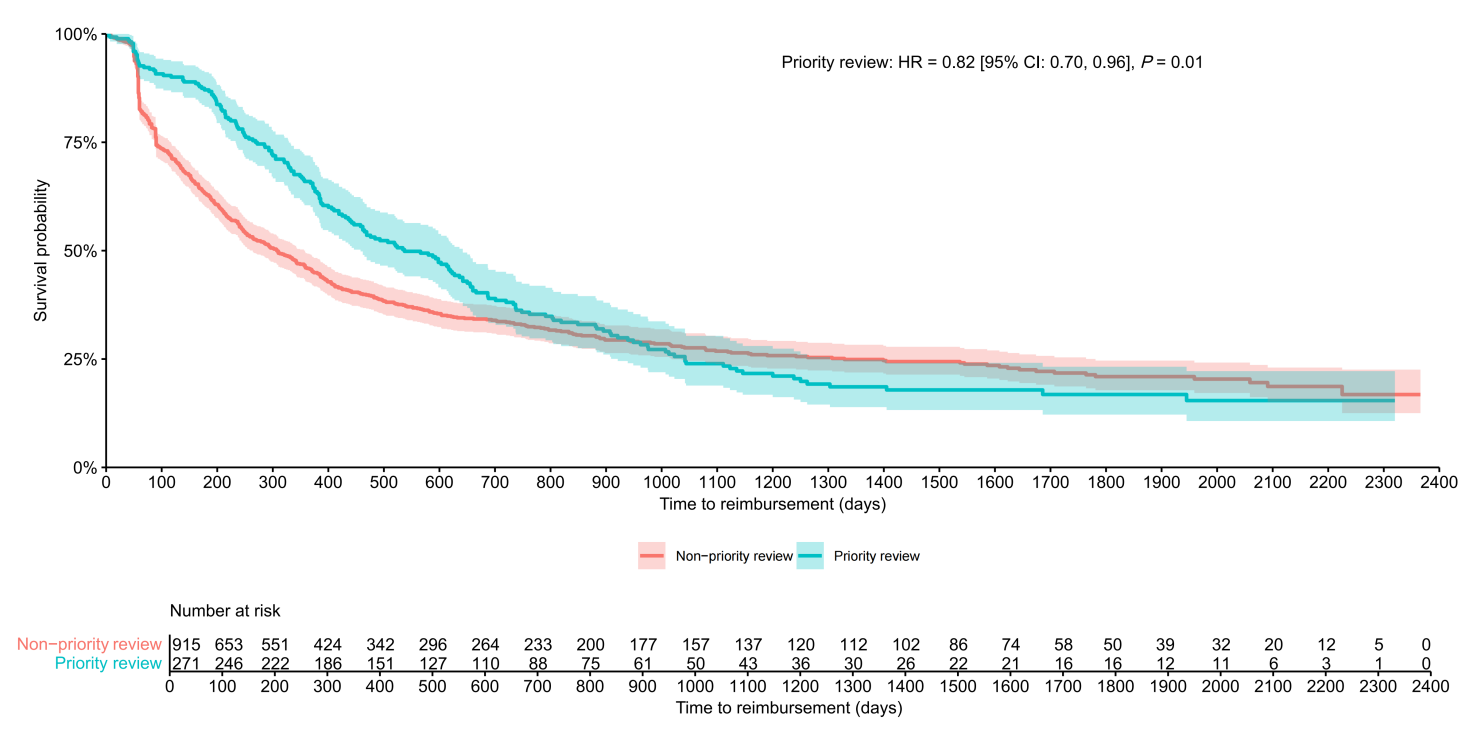


Figure S1: Kaplan–Meyer curves of public funding reimbursement and time to reimbursement (by priority review)

**Notes:** HR = hazard ratio; *P* for test of group difference; Bold means statistically significant.


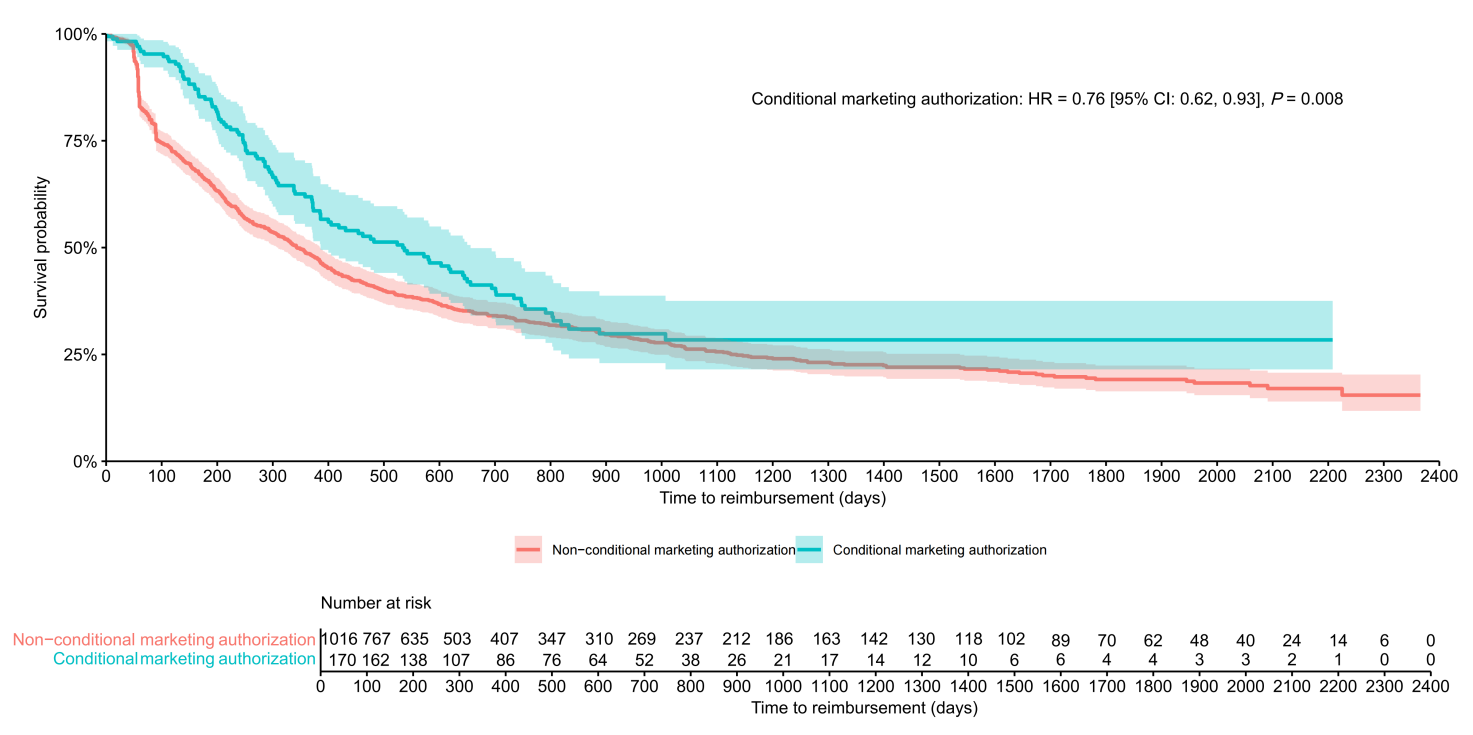


Figure S2: Kaplan–Meyer curves of public funding reimbursement and time to reimbursement (by conditional marketing authorization)

**Notes:** HR = hazard ratio; *P* for test of group difference; Bold means statistically significant.


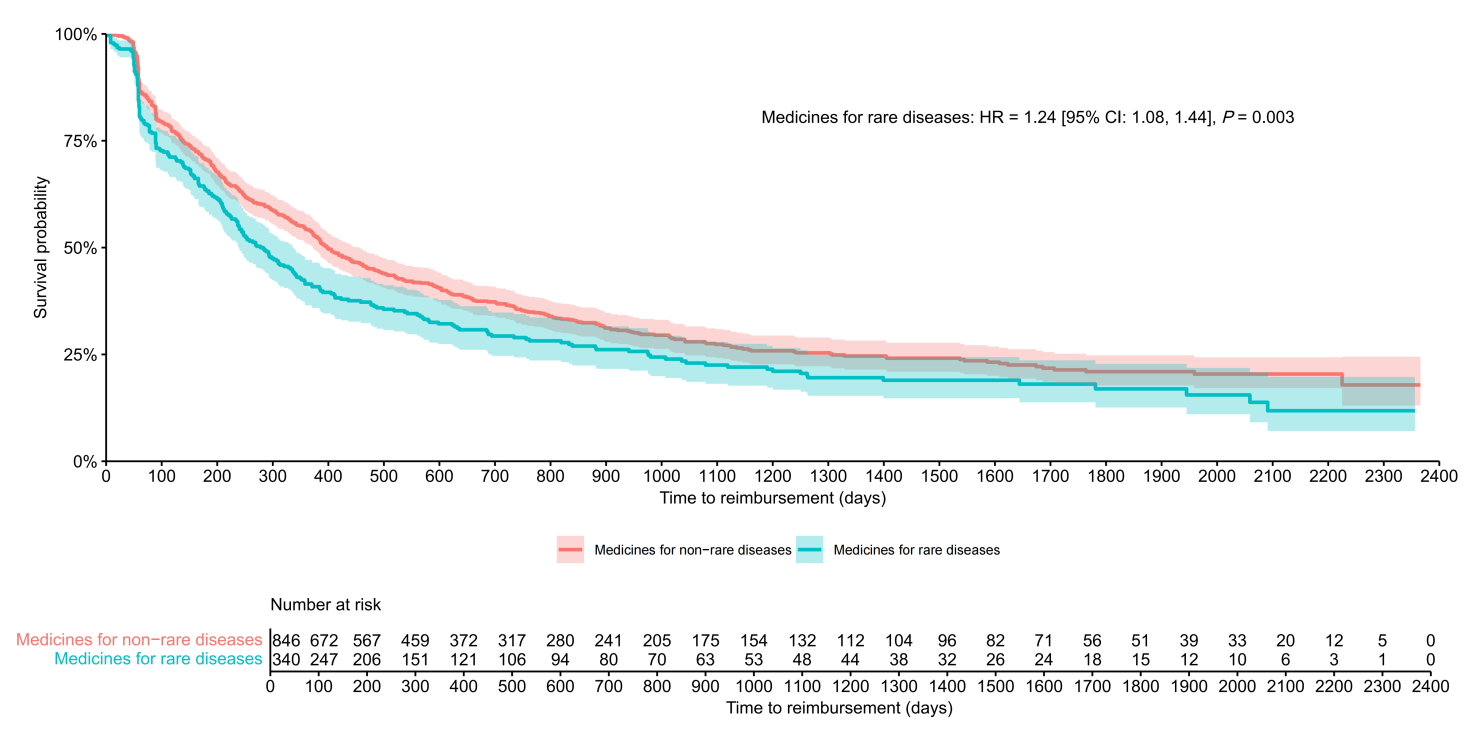


Figure S3: Kaplan–Meyer curves of public funding reimbursement and time to reimbursement (by medicines for rare diseases)

**Notes:** HR = hazard ratio; *P* for test of group difference; Bold means statistically significant.


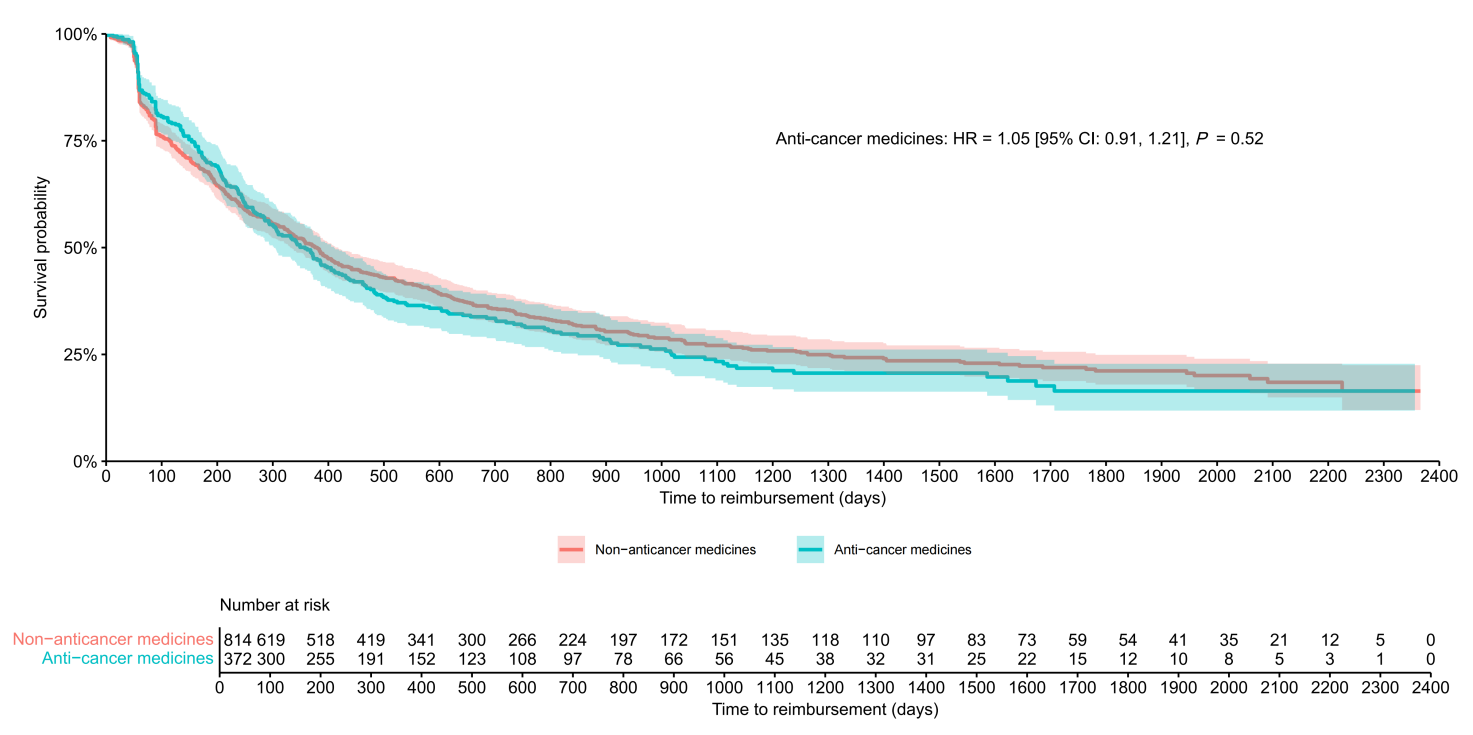


Figure S4: Kaplan–Meyer curves of public funding reimbursement and time to reimbursement (by anti-cancer medicines)

**Notes:** HR = hazard ratio; *P* for test of group difference; Bold means statistically significant.


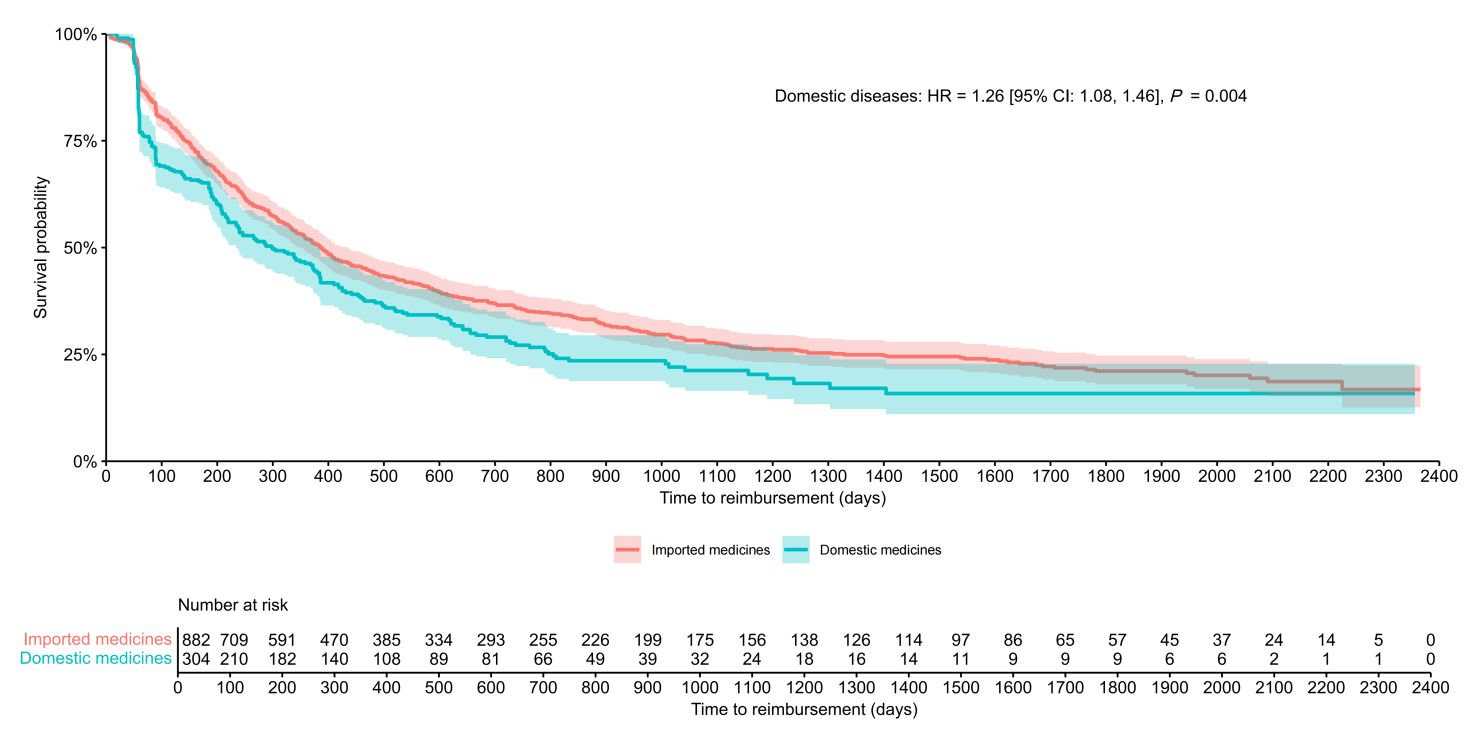


Figure S5: Kaplan–Meyer curves of public funding reimbursement and time to reimbursement (by origin)

**Notes:** HR = hazard ratio; *P* for test of group difference; Bold means statistically significant.


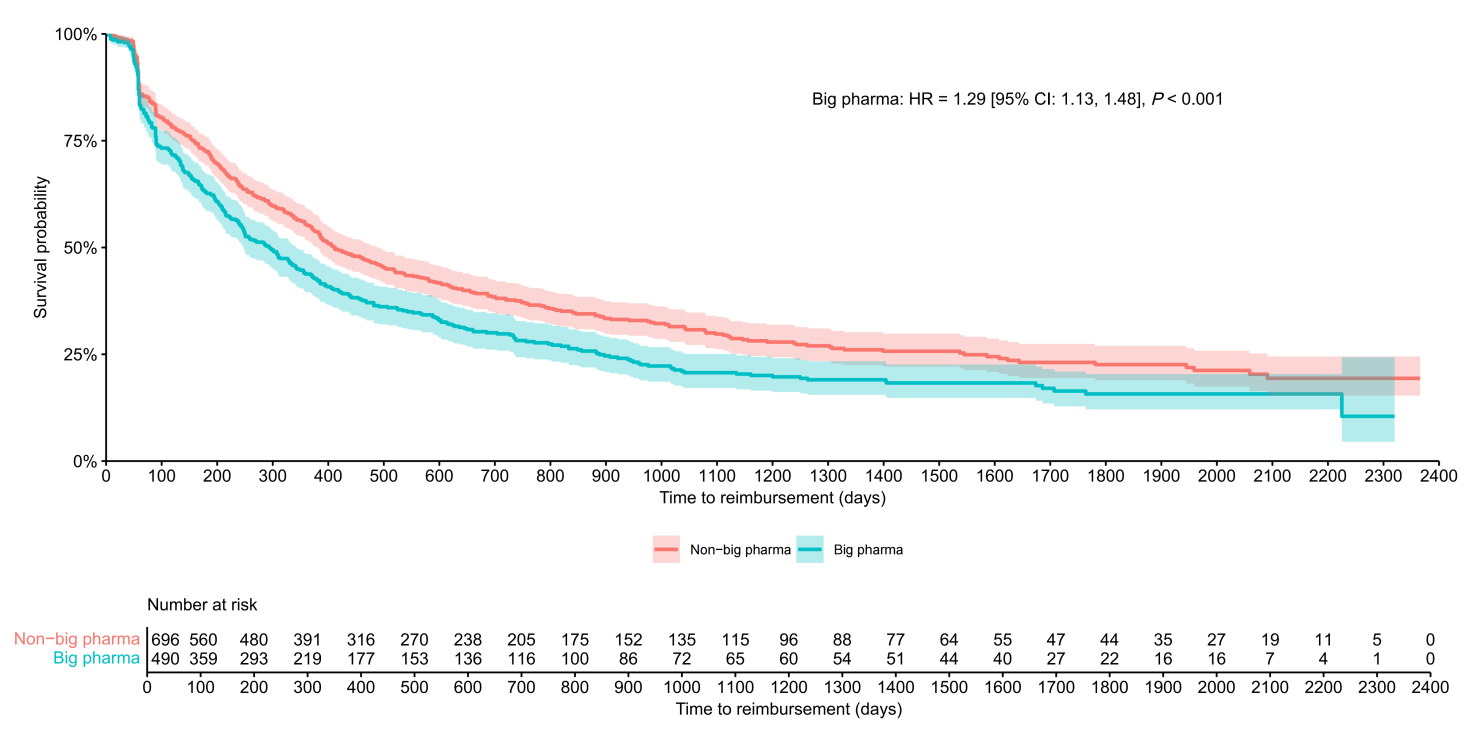


Figure S6: Kaplan–Meyer curves of public funding reimbursement and time to reimbursement (by big pharma)

**Notes:** HR = hazard ratio; *P* for test of group difference; Bold means statistically significant.


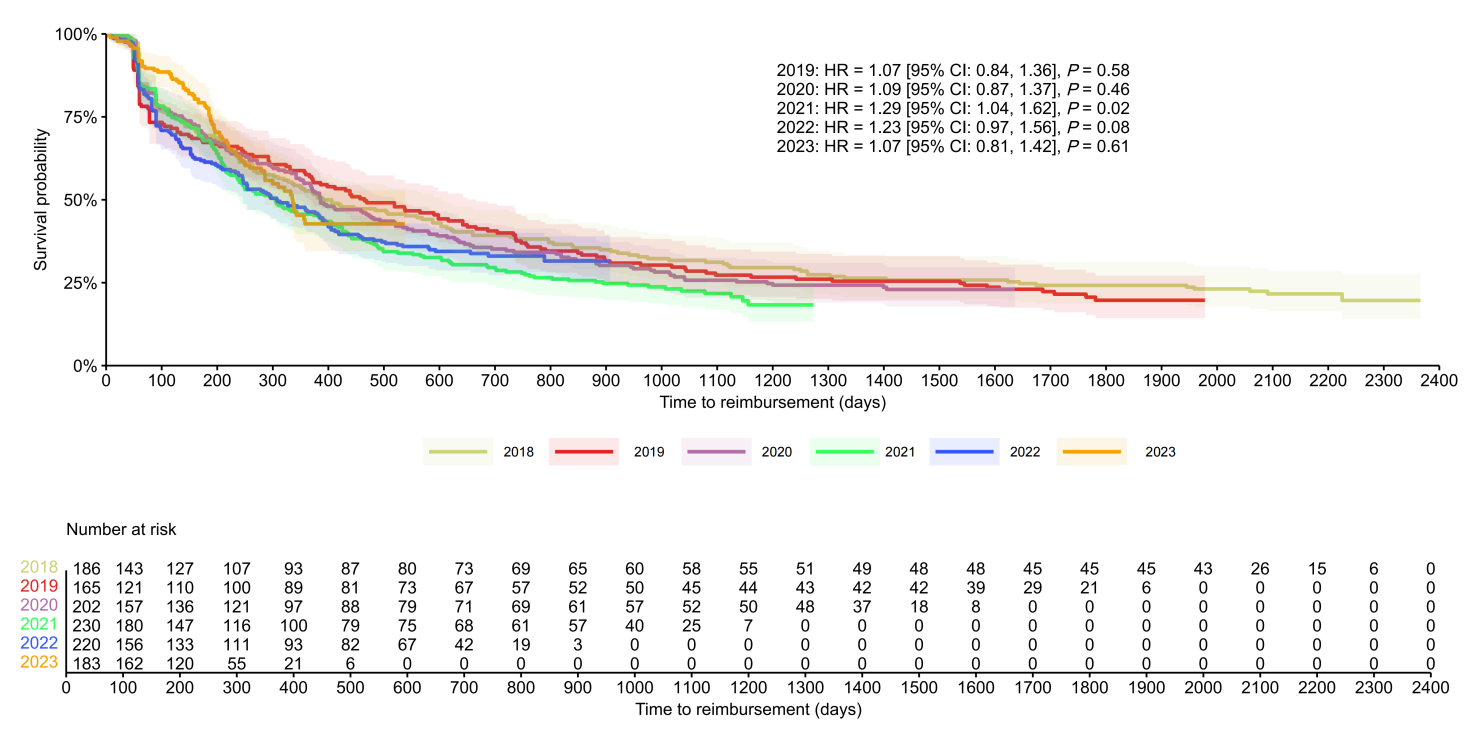


Figure S7: Kaplan–Meyer curves of public funding reimbursement and time to reimbursement (by year)

**Notes:** HR = hazard ratio; *P* for test of group difference; Bold means statistically significant.

Table S7: Univariate Cox proportional hazard regression analysis in different countries

| **Category** | **Variable** | **No. of medicines** | **Hazard Ratio** | **95% CI** | ***P***-value |
| --- | --- | --- | --- | --- | --- |
| **All countries** | Country |  |  |  |  |
|  | China (Ref.) | 300 |  |  |  |
|  | Japan | 226 | 9.11 | 7.38, 11.25 | **<0.001** |
|  | France | 233 | 1.59 | 1.29, 1.97 | **<0.001** |
|  | United Kingdom | 228 | 1.27 | 1.03, 1.57 | **0.03** |
|  | Switzerland | 199 | 1.21 | 0.96, 1.52 | 0.11 |
|  | Year |  |  |  |  |
|  | 2018 (Ref.) | 186 |  |  |  |
|  | 2019 | 165 | 1.07 | 0.84, 1.36 | 0.58 |
|  | 2020 | 202 | 1.09 | 0.87, 1.37 | 0.46 |
|  | 2021 | 230 | 1.29 | 1.04, 1.62 | **0.02** |
|  | 2022 | 220 | 1.23 | 0.97, 1.56 | 0.08 |
|  | 2023 | 183 | 1.07 | 0.81, 1.42 | 0.61 |
|  | Priority review |  |  |  |  |
|  | No (Ref.) | 915 |  |  |  |
|  | Yes | 271 | 0.82 | 0.70, 0.96 | **0.01** |
|  | Conditional marketing authorization | |  |  |  |
|  | No (Ref.) | 1016 |  |  |  |
|  | Yes | 170 | 0.76 | 0.62, 0.93 | **0.008** |
|  | Orphan medicines |  |  |  |  |
|  | No (Ref.) | 846 |  |  |  |
|  | Yes | 340 | 1.24 | 1.08, 1.44 | **0.003** |
|  | Anti-cancer medicines |  |  |  |  |
|  | No (Ref.) | 814 |  |  |  |
|  | Yes | 372 | 1.05 | 0.91, 1.21 | 0.52 |
|  | Origin |  |  |  |  |
|  | Imported (Ref.) | 882 |  |  |  |
|  | Domestic | 304 | 1.26 | 1.08, 1.46 | **0.004** |
|  | Big pharma |  |  |  |  |
|  | No (Ref.) | 696 |  |  |  |
|  | Yes | 490 | 1.29 | 1.13, 1.48 | **<0.001** |
| **China** | Year |  |  |  |  |
|  | 2018 (Ref.) | 47 |  |  |  |
|  | 2019 | 44 | 1.12 | 0.69, 1.83 | 0.64 |
|  | 2020 | 46 | 1.48 | 0.91, 2.43 | 0.12 |
|  | 2021 | 60 | 1.95 | 1.22, 3.11 | **0.005** |
|  | 2022 | 38 | 1.29 | 0.71, 2.34 | 0.39 |
|  | 2023 | 65 | 3.56 | 1.99, 6.35 | **<0.001** |
|  | Priority review |  |  |  |  |
|  | No (Ref.) | 109 |  |  |  |
|  | Yes | 191 | 1.10 | 0.81, 1.50 | 0.54 |
|  | Conditional marketing authorization | |  |  |  |
|  | No (Ref.) | 231 |  |  |  |
|  | Yes | 69 | 0.65 | 0.45, 0.96 | **0.03** |
|  | Orphan medicines |  |  |  |  |
|  | No (Ref.) | 264 |  |  |  |
|  | Yes | 36 | 1.15 | 0.76, 1.76 | 0.50 |
|  | Anti-cancer medicines |  |  |  |  |
|  | No (Ref.) | 186 |  |  |  |
|  | Yes | 114 | 0.81 | 0.60, 1.10 | 0.17 |
|  | Origin |  |  |  |  |
|  | Imported (Ref.) | 178 |  |  |  |
|  | Domestic | 122 | 1.97 | 1.47, 2.65 | **<0.001** |
|  | Big pharma |  |  |  |  |
|  | No (Ref.) | 191 |  |  |  |
|  | Yes | 109 | 0.79 | 0.59, 1.07 | 0.13 |
| **Japan** | Year |  |  |  |  |
|  | 2018 (Ref.) | 35 |  |  |  |
|  | 2019 | 37 | 1.44 | 0.90, 2.29 | 0.13 |
|  | 2020 | 37 | 1.25 | 0.78, 2.00 | 0.35 |
|  | 2021 | 47 | 0.86 | 0.54, 1.35 | 0.51 |
|  | 2022 | 46 | 0.94 | 0.60, 1.46 | 0.77 |
|  | 2023 | 24 | 0.34 | 0.18, 0.65 | **0.001** |
|  | Priority review |  |  |  |  |
|  | No (Ref.) | 209 |  |  |  |
|  | Yes | 17 | 1.94 | 1.18, 3.21 | **0.009** |
|  | Conditional marketing authorization | |  |  |  |
|  | No (Ref.) | 223 |  |  |  |
|  | Yes | 3 | 2.09 | 0.67, 6.57 | 0.21 |
|  | Orphan medicines |  |  |  |  |
|  | No (Ref.) | 152 |  |  |  |
|  | Yes | 74 | 1.50 | 1.13, 2.01 | **0.006** |
|  | Anti-cancer medicines |  |  |  |  |
|  | No (Ref.) | 169 |  |  |  |
|  | Yes | 57 | 1.27 | 0.93, 1.73 | 0.14 |
|  | Origin |  |  |  |  |
|  | Imported (Ref.) | 123 |  |  |  |
|  | Domestic | 103 | 1.15 | 0.88, 1.51 | 0.32 |
|  | Big pharma |  |  |  |  |
|  | No (Ref.) | 132 |  |  |  |
|  | Yes | 94 | 1.00 | 0.76, 1.32 | 0.98 |
| **France** | Year |  |  |  |  |
|  | 2018 (Ref.) | 38 |  |  |  |
|  | 2019 | 29 | 0.70 | 0.40, 1.23 | 0.22 |
|  | 2020 | 38 | 0.88 | 0.52, 1.50 | 0.64 |
|  | 2021 | 46 | 1.20 | 0.73, 1.95 | 0.47 |
|  | 2022 | 52 | 1.02 | 0.63, 1.67 | 0.92 |
|  | 2023 | 30 | 1.57 | 0.90, 2.75 | 0.11 |
|  | Priority review |  |  |  |  |
|  | No (Ref.) | 226 |  |  |  |
|  | Yes | 7 | 2.02 | 0.94, 4.31 | 0.07 |
|  | Conditional marketing authorization | |  |  |  |
|  | No (Ref.) | 199 |  |  |  |
|  | Yes | 34 | 1.23 | 0.81, 1.87 | 0.33 |
|  | Orphan medicines |  |  |  |  |
|  | No (Ref.) | 157 |  |  |  |
|  | Yes | 76 | 1.45 | 1.06, 1.99 | **0.02** |
|  | Anti-cancer medicines |  |  |  |  |
|  | No (Ref.) | 160 |  |  |  |
|  | Yes | 73 | 1.09 | 0.79, 1.51 | 0.61 |
|  | Origin |  |  |  |  |
|  | Imported (Ref.) | 223 |  |  |  |
|  | Domestic | 10 | 1.02 | 0.48, 2.18 | 0.96 |
|  | Big pharma |  |  |  |  |
|  | No (Ref.) | 134 |  |  |  |
|  | Yes | 99 | 1.43 | 1.05, 1.93 | **0.02** |
| **United Kingdom** | Year |  |  |  |  |
|  | 2018 (Ref.) | 38 |  |  |  |
|  | 2019 | 29 | 1.05 | 0.60, 1.84 | 0.87 |
|  | 2020 | 43 | 1.01 | 0.60, 1.71 | 0.97 |
|  | 2021 | 44 | 1.81 | 1.08, 3.01 | **0.02** |
|  | 2022 | 44 | 1.63 | 0.94, 2.82 | 0.08 |
|  | 2023 | 30 | 2.43 | 1.26, 4.70 | **0.008** |
|  | Priority review |  |  |  |  |
|  | No (Ref.) | 188 |  |  |  |
|  | Yes | 40 | 1.55 | 1.06, 2.26 | **0.02** |
|  | Conditional marketing authorization | |  |  |  |
|  | No (Ref.) | 199 |  |  |  |
|  | Yes | 29 | 1.88 | 1.21, 2.92 | **0.005** |
|  | Orphan medicines |  |  |  |  |
|  | No (Ref.) | 158 |  |  |  |
|  | Yes | 70 | 0.99 | 0.71, 1.39 | 0.96 |
|  | Anti-cancer medicines |  |  |  |  |
|  | No (Ref.) | 160 |  |  |  |
|  | Yes | 68 | 2.01 | 1.45, 2.80 | **<0.001** |
|  | Origin |  |  |  |  |
|  | Imported (Ref.) | 206 |  |  |  |
|  | Domestic | 22 | 0.71 | 0.40, 1.25 | 0.23 |
|  | Big pharma |  |  |  |  |
|  | No (Ref.) | 138 |  |  |  |
|  | Yes | 90 | 1.71 | 1.24, 2.34 | **0.001** |
| **Switzerland** | Year |  |  |  |  |
|  | 2018 (Ref.) | 28 |  |  |  |
|  | 2019 | 26 | 1.53 | 0.80, 2.95 | 0.20 |
|  | 2020 | 38 | 1.34 | 0.73, 2.45 | 0.34 |
|  | 2021 | 33 | 1.18 | 0.62, 2.24 | 0.62 |
|  | 2022 | 40 | 1.06 | 0.55, 2.04 | 0.86 |
|  | 2023 | 34 | 0.88 | 0.40, 1.91 | 0.74 |
|  | Priority review |  |  |  |  |
|  | No (Ref.) | 183 |  |  |  |
|  | Yes | 16 | 1.75 | 1.01, 3.06 | 0.051 |
|  | Conditional marketing authorization | |  |  |  |
|  | No (Ref.) | 164 |  |  |  |
|  | Yes | 35 | 1.02 | 0.64, 1.63 | 0.92 |
|  | Orphan medicines |  |  |  |  |
|  | No (Ref.) | 115 |  |  |  |
|  | Yes | 84 | 0.93 | 0.65, 1.33 | 0.68 |
|  | Anti-cancer medicines |  |  |  |  |
|  | No (Ref.) | 139 |  |  |  |
|  | Yes | 60 | 1.18 | 0.81, 1.72 | 0.39 |
|  | Origin |  |  |  |  |
|  | Imported (Ref.) | 152 |  |  |  |
|  | Domestic | 47 | 0.59 | 0.37, 0.93 | **0.02** |
|  | Big pharma |  |  |  |  |
|  | No (Ref.) | 101 |  |  |  |
|  | Yes | 98 | 2.07 | 1.44, 2.99 | **<0.001** |

**Notes:** CI = confidence interval; *P*-value for test of group difference; Bold means statistically significant.

Table S8: M**ultiple** Cox proportional hazard regression analysis in different countries

|  | HR (95%CI) | | | | | |
| --- | --- | --- | --- | --- | --- | --- |
|  | All countries | China | Japan | France | United Kingdom | Switzerland |
| **Priority review** | 1.45 (1.17, 1.79) ** | 1.48 (1.05, 2.09) * | 1.58 (0.91, 2.74) | 1.93 (0.84, 4.48) | 1.5 (1.01, 2.22) * | 1.45 (0.79, 2.67) |
| **Conditional marketing authorization** | 0.87 (0.69, 1.09) | 0.42 (0.27, 0.68) *** | 1.71 (0.51, 5.74) | 1.04 (0.64, 1.70) | 1.18 (0.71, 1.95) | 0.88 (0.52, 1.49) |
| **Medicines for rare diseases** | 1.29 (1.11, 1.51) ** | 0.93 (0.58, 1.48) | 1.52 (1.11, 2.08) ** | 1.45 (1.04, 2.04) * | 1.15 (0.80, 1.65) | 0.85 (0.58, 1.25) |
| **Anti-cancer medicines** | 1.18 (1.01, 1.38) * | 0.87 (0.62, 1.23) | 1.09 (0.77, 1.53) | 0.99 (0.68, 1.43) | 1.91 (1.34, 2.71) *** | 1.01 (0.67, 1.53) |
| **Domestic medicines** | 1.24 (1.04, 1.47) * | 2.09 (1.43, 3.06) *** | 1.19 (0.82, 1.72) | 1.13 (0.52, 2.46) | 0.59 (0.33, 1.06) | 0.61 (0.38, 0.98) * |
| **Big pharma** | 1.38 (1.19, 1.60) *** | 1.21 (0.83, 1.78) | 1.18 (0.81, 1.74) | 1.49 (1.08, 2.06) * | 1.80 (1.29, 2.52) *** | 2.01 (1.36, 2.99) *** |
| **Year (2018 as reference)** |  |  |  |  |  |  |
| 2019 | 1.11 (0.87, 1.41) | 1.18 (0.72, 1.93) | 1.46 (0.91, 2.34) | 0.91 (0.50, 1.66) | 1.01 (0.56, 1.81) | 1.04 (0.52, 2.08) |
| 2020 | 1.08 (0.86, 1.36) | 1.55 (0.94, 2.55) | 1.41 (0.88, 2.28) | 0.87 (0.49, 1.54) | 0.98 (0.57, 1.66) | 1.06 (0.55, 2.01) |
| 2021 | 1.07 (0.85, 1.35) | 2.34 (1.41, 3.87) ** | 0.86 (0.54, 1.37) | 1.36 (0.80, 2.29) | 1.68 (0.99, 2.85) | 1.06 (0.54, 2.08) |
| 2022 | 1.15 (0.90, 1.46) | 1.66 (0.88, 3.12) | 1.01 (0.64, 1.59) | 1.16 (0.69, 1.95) | 1.65 (0.95, 2.87) | 0.97 (0.48, 1.95) |
| 2023 | 0.97 (0.73, 1.28) | 4.28 (2.31, 7.90) *** | 0.38 (0.19, 0.73) ** | 1.75 (0.97, 3.17) | 2.38 (1.19, 4.74) * | 0.72 (0.33, 1.60) |
| **Country (China as reference)** |  |  |  |  |  |  |
| Japan | 11.29 (8.63, 14.77) *** |  |  |  |  |  |
| France | 2.08 (1.60, 2.72) *** |  |  |  |  |  |
| United Kingdom | 1.55 (1.21, 1.99) ** |  |  |  |  |  |
| Switzerland | 1.37 (1.04, 1.8) * |  |  |  |  |  |

**Notes:** HR = hazard ratio; CI = confidence interval; *P*-value for test of group difference: **P*-value < 0.05; ***P*-value < 0.01; ****P*-value < 0.001.


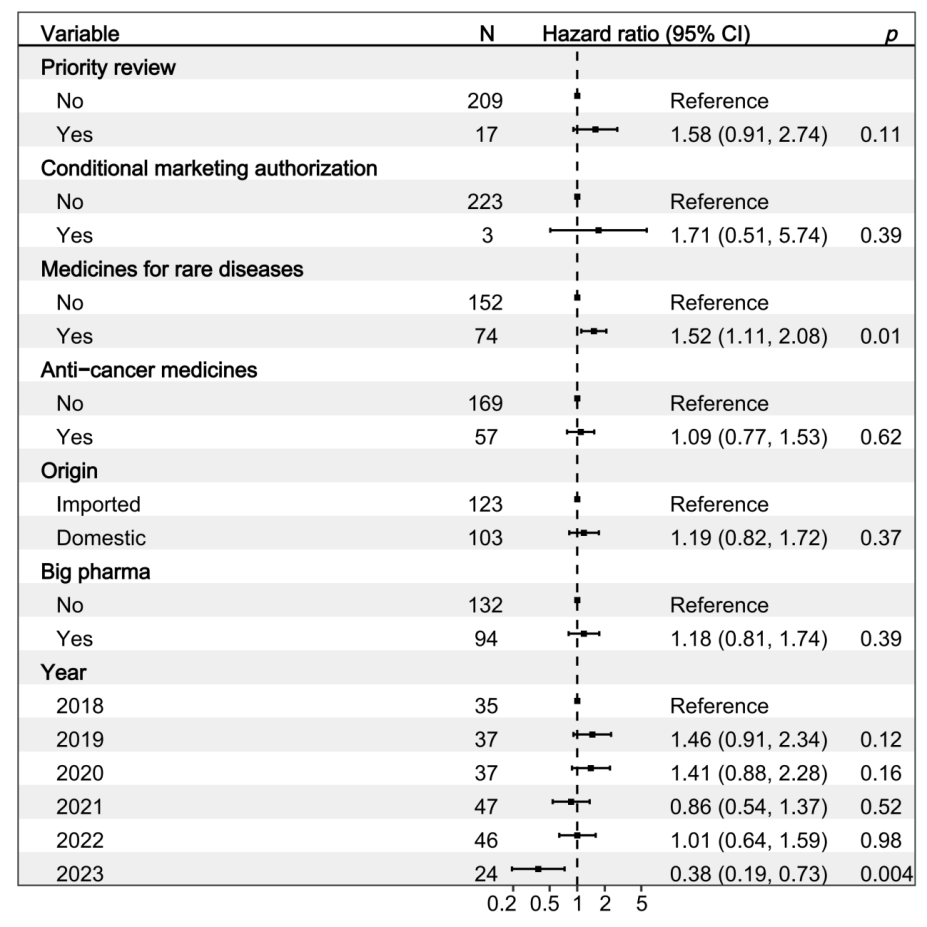


Figure S8: Hazard Ratios for public funding reimbursement of novel medicines in Japan

**Notes:** CI = confidence interval


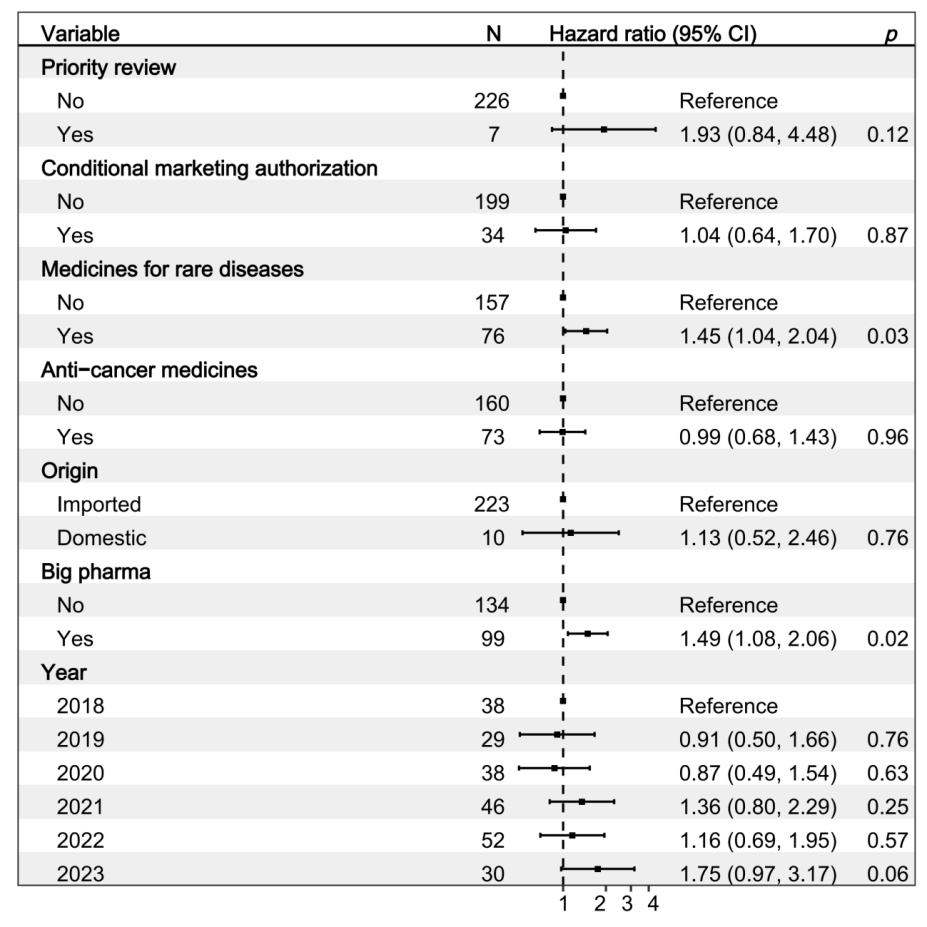


Figure S9: Hazard Ratios for public funding reimbursement of novel medicines in France

**Notes:** CI = confidence interval


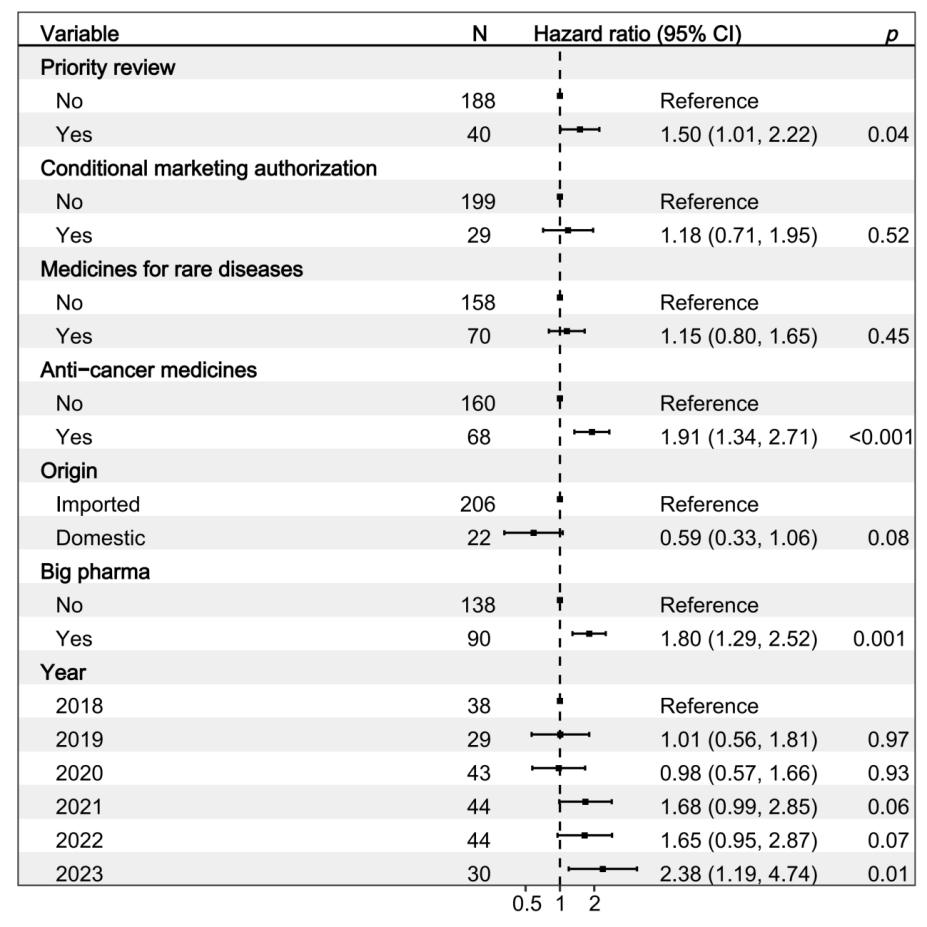


Figure S10: Hazard Ratios for public funding reimbursement of novel medicines in United Kingdom

**Notes:** CI = confidence interval


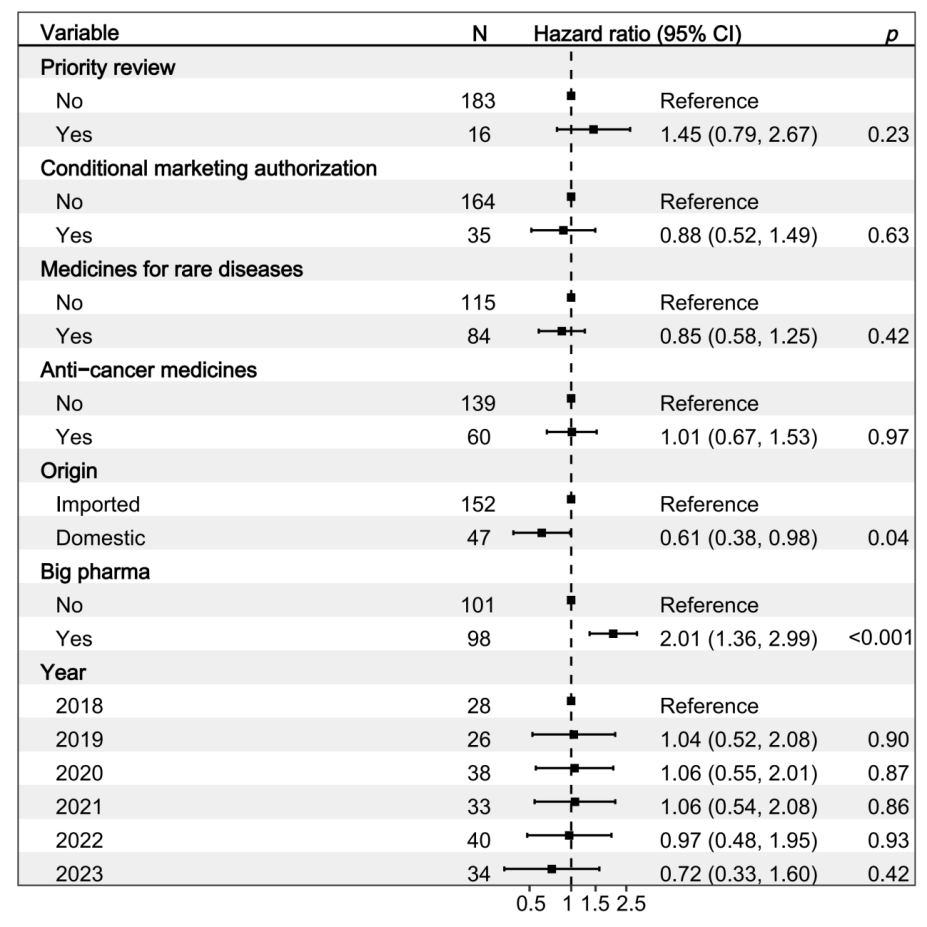


Figure S11: Hazard Ratios for public funding reimbursement of novel medicines in Switzerland

**Notes:** CI = confidence interval


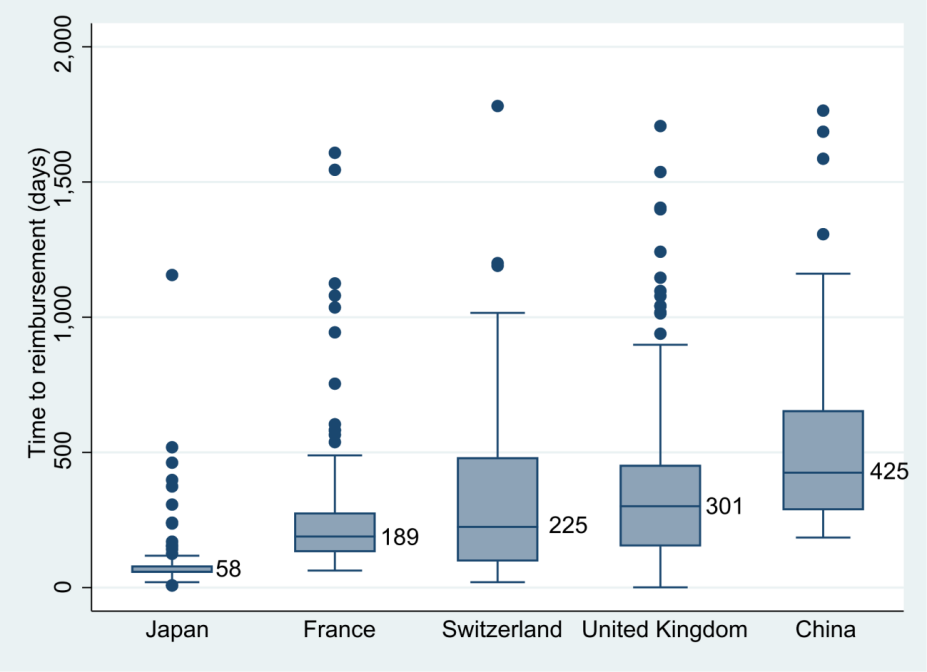


Figure S12: Sensitivity analysis of boxplots of time to reimbursement for the reimbursed novel medicines in five countries


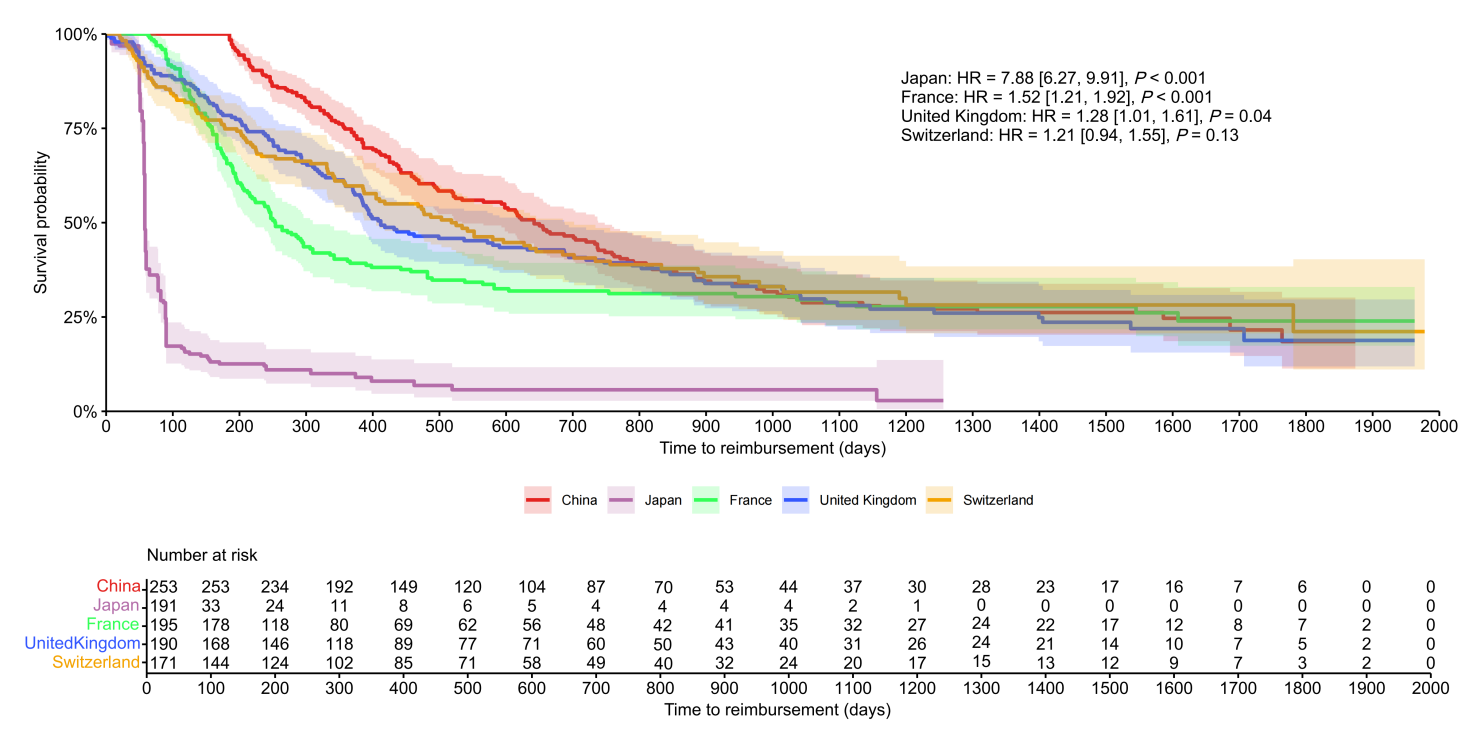


Figure S13: Sensitivity analysis of Kaplan-Meyer curve of time to reimbursement in five countries

Table S9: Sensitivity analysis of Kruskal-Wallis H test for difference in TTR between the countries

|  | **China** | **Japan** | **France** | **United Kingdom** | **Switzerland** |
| --- | --- | --- | --- | --- | --- |
| **China** |  |  |  |  |  |
| **Japan** | **<0.000001** |  |  |  |  |
| **France** | **<0.000001** | **<0.000001** |  |  |  |
| **United Kingdom** | **0.002** | **<0.000001** | **0.03** |  |  |
| **Switzerland** | **0.0001** | **<0.000001** | 0.16 | 0.19 |  |

**Notes:** Bold means statistically significant.

Table S10: Sensitivity analysis of Kruskal-Wallis H test with B**onferroni correction** for difference in TTR between the countries

|  | **China** | **Japan** | **France** | **United Kingdom** | **Switzerland** |
| --- | --- | --- | --- | --- | --- |
| **China** |  |  |  |  |  |
| **Japan** | **<0**.**001** |  |  |  |  |
| **France** | **0**.**03** | **<0.001** |  |  |  |
| **United Kingdom** | >0.99 | **<0.001** | 0.97 |  |  |
| **Switzerland** | 0.22 | **<0.001** | >0.99 | >0.99 |  |

**Notes:** Bold means statistically significant. Given the number of statistical tests performed within this study we used a Bonferroni correction. For this correction we corrected for individual comparisons between countries. The correction used for the paired differences between the countries was the number of individual paired comparisons (n = 10).

Table S11: Sensitivity analysis of univariate Cox proportional hazard regression analysis in all countries and China

| **Categories** | **Variables** | **No. of medicines** | **Hazard Ratio** | **95% CI** | ***P*-value** |
| --- | --- | --- | --- | --- | --- |
| **All countries** | Country |  |  |  |  |
|  | China (Ref.) | 253 |  |  |  |
|  | Japan | 191 | 7.88 | 6.27, 9.91 | **<0.001** |
|  | France | 195 | 1.52 | 1.21, 1.92 | **<0.001** |
|  | United Kingdom | 190 | 1.28 | 1.01, 1.61 | **0.04** |
|  | Switzerland | 171 | 1.21 | 0.94, 1.55 | 0.13 |
|  | Year |  |  |  |  |
|  | 2019 (Ref.) | 165 |  |  |  |
|  | 2020 | 202 | 1.04 | 0.82, 1.31 | 0.76 |
|  | 2021 | 230 | 1.23 | 0.98, 1.54 | 0.08 |
|  | 2022 | 220 | 1.17 | 0.92, 1.49 | 0.21 |
|  | 2023 | 183 | 1.03 | 0.77, 1.36 | 0.85 |
|  | Priority review |  |  |  |  |
|  | No (Ref.) | 779 |  |  |  |
|  | Yes | 221 | 0.83 | 0.70, 0.99 | **0.04** |
|  | Conditional marketing authorization |  |  |  |  |
|  | No (Ref.) | 835 |  |  |  |
|  | Yes | 165 | 0.76 | 0.62, 0.93 | **0.009** |
|  | Medicines for rare diseases |  |  |  |  |
|  | No (Ref.) | 715 |  |  |  |
|  | Yes | 285 | 1.27 | 1.08, 1.49 | **0.004** |
|  | Anti-cancer medicines |  |  |  |  |
|  | No (Ref.) | 679 |  |  |  |
|  | Yes | 321 | 1.01 | 0.86, 1.18 | 0.93 |
|  | Origin |  |  |  |  |
|  | Imported (Ref.) | 735 |  |  |  |
|  | Domestic | 265 | 1.22 | 1.04, 1.45 | **0.02** |
|  | Big pharma |  |  |  |  |
|  | No (Ref.) | 596 |  |  |  |
|  | Yes | 404 | 1.26 | 1.09, 1.46 | **0.002** |
| **China** | Year |  |  |  |  |
|  | 2019 (Ref.) | 44 |  |  |  |
|  | 2020 | 46 | 1.35 | 0.83, 2.21 | 0.23 |
|  | 2021 | 60 | 1.72 | 1.08, 2.73 | **0.02** |
|  | 2022 | 38 | 1.13 | 0.63, 2.05 | 0.68 |
|  | 2023 | 65 | 3.02 | 1.69, 5.39 | **<0.001** |
|  | Priority review |  |  |  |  |
|  | No (Ref.) | 97 |  |  |  |
|  | Yes | 156 | 1.11 | 0.79, 1.55 | 0.55 |
|  | Conditional marketing authorization |  |  |  |  |
|  | No (Ref.) | 188 |  |  |  |
|  | Yes | 65 | 0.65 | 0.44, 0.97 | **0.03** |
|  | Medicines for rare diseases |  |  |  |  |
|  | No (Ref.) | 221 |  |  |  |
|  | Yes | 32 | 1.10 | 0.69, 1.74 | 0.69 |
|  | Anti-cancer medicines |  |  |  |  |
|  | No (Ref.) | 158 |  |  |  |
|  | Yes | 95 | 0.74 | 0.53, 1.03 | 0.08 |
|  | Origin |  |  |  |  |
|  | Imported (Ref.) | 142 |  |  |  |
|  | Domestic | 111 | 1.84 | 1.33, 2.54 | **<0.001** |
|  | Big pharma |  |  |  |  |
|  | No (Ref.) | 168 |  |  |  |
|  | Yes | 85 | 0.84 | 0.60, 1.18 | 0.32 |

**Notes:** CI = confidence interval; *P*-value for test of group difference; Bold means statistically significant.

**Table S12:** **Sensitivity analysis of multiple Cox proportional hazard regression analysis in all countries and China**

|  | **All countries** | | |  | **China** | | |
| --- | --- | --- | --- | --- | --- | --- | --- |
| **Variables** | **Hazard Ratio** | **95% CI** | ***P*-value** |  | **Hazard Ratio** | **95% CI** | ***P*-value** |
| **Year** |  |  |  |  |  |  |  |
| 2020 | 0.99 | 0.78, 1.25 | 0.92 |  | 1.35 | 0.82, 2.23 | 0.24 |
| 2021 | 1.00 | 0.79, 1.27 | 0.997 |  | 2.01 | 1.21, 3.34 | **0.007** |
| 2022 | 1.05 | 0.82, 1.35 | 0.68 |  | 1.40 | 0.74, 2.64 | 0.30 |
| 2023 | 0.91 | 0.68, 1.21 | 0.52 |  | 3.54 | 1.89, 6.63 | **<0.001** |
| **Priority review** |  |  |  |  |  |  |  |
| No (Ref.) |  |  |  |  |  |  |  |
| Yes | 1.45 | 1.14, 1.84 | **0.002** |  | 1.51 | 1.03, 2.22 | **0.04** |
| **Conditional marketing authorization** | |  |  |  |  |  |  |
| No (Ref.) |  |  |  |  |  |  |  |
| Yes | 0.89 | 0.70, 1.12 | 0.32 |  | 0.47 | 0.29, 0.76 | **0.002** |
| **Orphan medicines** |  |  |  |  |  |  |  |
| No (Ref.) |  |  |  |  |  |  |  |
| Yes | 1.29 | 1.09, 1.53 | **0.003** |  | 0.86 | 0.51, 1.44 | 0.56 |
| **Anti-cancer medicines** | |  |  |  |  |  |  |
| No (Ref.) |  |  |  |  |  |  |  |
| Yes | 1.13 | 0.96, 1.35 | 0.15 |  | 0.76 | 0.52, 1.12 | 0.17 |
| **Origin** |  |  |  |  |  |  |  |
| Imported (Ref.) |  |  |  |  |  |  |  |
| Domestic | 1.23 | 1.02, 1.49 | **0.03** |  | 2.14 | 1.36, 3.36 | **0.001** |
| **Big pharma** |  |  |  |  |  |  |  |
| No (Ref.) |  |  |  |  |  |  |  |
| Yes | 1.34 | 1.14, 1.58 | **<0.001** |  | 1.32 | 0.83, 2.10 | 0.25 |
| **Country** |  |  |  |  |  |  |  |
| China (Ref.) |  |  |  |  |  |  |  |
| Japan | 9.65 | 7.23, 12.9 | **<0.001** |  |  |  |  |
| France | 1.98 | 1.48, 2.67 | **<0.001** |  |  |  |  |
| United Kingdom | 1.55 | 1.18, 2.03 | **0.002** |  |  |  |  |
| Switzerland | 1.34 | 0.99, 1.80 | 0.06 |  |  |  |  |
|  | *N* = 1000 | | |  | *N* = 253 | | |

**Notes:** HR = hazard ratio; CI = confidence interval; *P*-value for test of group difference; Bold means statistically significant.

Table S13: Cross-country comparisons of mechanisms for reimbursement decision on novel medicines

|  | **China** | **Japan** | **France** | **United Kingdom** | **Switzerland** |
| --- | --- | --- | --- | --- | --- |
| Dominated health security system | Basic Health Insurance | Social Health Insurance | Mandatory Social Health Insurance | National Health Service | Mandatory health insurance managed in the non-profit part of commercial insurers under strict government regulation |
| Centralized reimbursement list | Yes | Yes | Separate lists for outpatient and inpatient | Yes (Through inclusion in the published Guidance) | Separate lists for outpatient and inpatient |
| Update frequency | Once per year | Four times per year | Once per year | As needed | Once per year |
| Centralized health technology assessment agency | No | Health Technology Assessment Center for Health and Medical Economics | Transparency Committee | National Institute for Health and Care Excellence | No |
| Criteria for public funding reimbursement | Efficacy, safety, innovativeness, equity and cost-effectiveness | Improvement in treatment, disease severity, clinical outcome including efficacy and safety, therapeutic class, availability of alternative therapies, public health impact | Clinical benefit and improvement of clinical benefit comparing with existing therapeutics | Clinical efficacy, cost-effectiveness, budget impact | Clinical efficacy, appropriateness, and economic benefit |

**Appendix: The list of big pharma (sort by alphabet)**

AbbVie Inc., Amgen Inc., AstraZeneca PLC, Bayer AG, Bristol-Myers Squibb Company, Eli Lilly and Company, Gilead Sciences, Inc., GlaxoSmithKline plc (GSK), Johnson & Johnson, Merck & Co., Inc., Novo Nordisk A/S, Novartis International AG, Pfizer Inc., Roche Holding AG, Sanofi S.A., Takeda Pharmaceutical Company Limited.

Reference

[1] U.S. Food and Drug Administration. New Drugs at FDA: CDER’s New Molecular Entities and New Therapeutic Biological Products [Internet]. <https://www.fda.gov/drugs/development-approval-process-drugs/new-drugs-fda-cders-new-molecular-entities-and-new-therapeutic-biological-products>. Accessed 3 Jun 2025.

[2] State Administration for Market Regulation. Provisions for Drug Registration. <https://www.gov.cn/zhengce/zhengceku/2020-04/01/content_5498012.htm>. Accessed 3 Jun 2025.

[3] Pharmaceuticals and Medical Devices Agency. New Pharmaceuticals (Prescription Drugs). <https://www.pmda.go.jp/review-services/drug-reviews/about-reviews/p-drugs/0021.html>. Accessed 3 Jun 2025.

[4] European Medicines Agency. Innovative medicine. <https://www.ema.europa.eu/en/glossary/innovative-medicine>. Accessed 3 Jun 2025.

[5] Erice statement on drug innovation. Br J Clin Pharmacol. 2008;65(3):440-1. doi:10.1111/j.1365-2125.2007.03033.x.

[6] Ward DJ, Martino OI, Simpson S, Stevens AJ. Decline in new drug launches: myth or reality? Retrospective observational study using 30 years of data from the UK. BMJ Open. 2013;3(2):e002088. doi:10.1136/bmjopen-2012-002088.

[7] Swiss Agency for Therapeutic Products. Federal Act on Medicinal Products and Medical Devices (Therapeutic Products Act, TPA). <https://www.fedlex.admin.ch/eli/cc/2001/422/en>. Accessed 3 Jun 2025.

[8] Center for Drug Evaluation, National Medical Products Administration. 2023 Annual Drug Review Report. <https://www.nmpa.gov.cn/xxgk/fgwj/gzwj/gzwjyp/20240204154334141.html>. Accessed 3 Jun 2025.

[9] Center for Drug Evaluation, National Medical Products Administration. 2022 Annual Drug Review Report. <https://www.nmpa.gov.cn/xxgk/fgwj/gzwj/gzwjyp/20230906163722146.html>. Accessed 3 Jun 2025.

[10] Center for Drug Evaluation, National Medical Products Administration. 2021 Annual Drug Review Report. <https://www.nmpa.gov.cn/directory/web/nmpa/xxgk/fgwj/gzwj/gzwjyp/20220601110541120.html>. Accessed 3 Jun 2025.

[11] Center for Drug Evaluation, National Medical Products Administration. 2020 Annual Drug Review Report. <https://www.nmpa.gov.cn/directory/web/nmpa/xxgk/fgwj/gzwj/gzwjyp/20210621142436183.html>. Accessed 3 Jun 2025.

[12] Center for Drug Evaluation, National Medical Products Administration. 2019 Annual Drug Review Report. <https://www.nmpa.gov.cn/xxgk/fgwj/gzwj/gzwjyp/20200731114330106.html>. Accessed 3 Jun 2025.

[13] Center for Drug Evaluation, National Medical Products Administration. 2018 Annual Drug Review Report. <https://www.nmpa.gov.cn/directory/web/nmpa/xxgk/fgwj/gzwj/gzwjyp/20190701175801236.html>. Accessed 3 Jun 2025.

[14] Global Approved New Drugs. Beijing, China. 2025. <https://data.pharmacodia.com/innovativeDrugs>. Accessed 3 Jun 2025.

[15] China Approved Drug System. Shanghai, China. 2025. <https://webvpn.cpu.edu.cn/https/77726476706e69737468656265737421e6fe51d23e31674a76468aa395/cfdadrug/list>. Accessed 3 Jun 2025.

[16] Global New Drug Database. Hangzhou, China. 2025. <https://db.dxy.cn/v5/globaldrugproject>. Accessed 3 Jun 2025.

[17] Pharmaceuticals and Medical Devices Agency. List of Approved Products. <https://www.pmda.go.jp/english/review-services/reviews/approved-information/drugs/0002.html>. Accessed 3 Jun 2025.

[18] Japan Approved Drug System. Shanghai, China. 2025. <https://webvpn.cpu.edu.cn/https/77726476706e69737468656265737421e6fe51d23e31674a76468aa395/pmda>. Accessed 3 Jun 2025.

[19] European Medicines Agency. Medicines. <https://www.ema.europa.eu/en/medicines/download-medicine-data>. Accessed 3 Jun 2025.

[20] Lara J, Bujar M, Kermad A, McAuslane N, Somuyiwa A. List of NASs approved by the six regulatory authorities. London, United Kingdom: Center for Innovation in Regulatory Science; 2024.

[21] Lara J, Bujar M, Kermad A, McAuslane N, Somuyiwa A. List of NASs approved by the six regulatory authorities. London, United Kingdom: Center for Innovation in Regulatory Science; 2023.

[22] Lara J, Bujar M, McAuslane N. New Active Substances (NASs) approved by six major authorities in 2021. London, United Kingdom: Center for Innovation in Regulatory Science; 2022.

[23] Bujar M, McAuslane N. New Active Substances (NASs) approved by six major authorities in 2020. London, United Kingdom: Center for Innovation in Regulatory Science; 2021.

[24] Rodier C, Bujar M, McAuslane N, Liberti L, Munro JW. New Active Substances (NASs) approved by six major authorities in 2019. London, United Kingdom: Center for Innovation in Regulatory Science; 2020.

[25] EMA Approved Drug System. Shanghai, China. 2025. <https://webvpn.cpu.edu.cn/https/77726476706e69737468656265737421e6fe51d23e31674a76468aa395/epyp/list>. Accessed 3 Jun 2025.

[26] Medicines and Healthcare products Regulatory Agency. Marketing authorisations: lists of granted licences. <https://www.gov.uk/government/collections/marketing-authorisations-lists-of-granted-licences>. Accessed 3 Jun 2025.

[27] UK Approved Drug System. Shanghai, China. 2025. <https://webvpn.cpu.edu.cn/https/77726476706e69737468656265737421e6fe51d23e31674a76468aa395/uk>. Accessed 3 Jun 2025.

[28] Swiss Agency for Therapeutic Products. List of authorised medicinal products. <https://www.swissmedic.ch/swissmedic/en/home/services/listen_neu.html>. Accessed 3 Jun 2025.

[29] Swiss Agency for Therapeutic Products. Swissmedic Annual Report 2018. <https://www.swissmedic.ch/swissmedic/en/home/about-us/publications/aktueller-geschaeftsbericht/swissmedic-geschaeftsbericht-2018.html>. Accessed 3 Jun 2025.

[30] Swiss Agency for Therapeutic Products. Swissmedic Annual Report 2019. <https://www.swissmedic.ch/swissmedic/en/home/about-us/publications/aktueller-geschaeftsbericht/swissmedic-geschaeftsbericht-2019.html>. Accessed 3 Jun 2025.

[31] Swiss Agency for Therapeutic Products. Swissmedic Annual Report 2020. <https://www.swissmedic.ch/swissmedic/en/home/about-us/publications/aktueller-geschaeftsbericht/swissmedic-geschaeftsbericht-2020.html>. Accessed 3 Jun 2025.

[32] Swiss Agency for Therapeutic Products. Swissmedic Annual Report 2021. <https://www.swissmedic.ch/swissmedic/en/home/about-us/publications/aktueller-geschaeftsbericht/swissmedic-geschaeftsbericht-2021.html>. Accessed 3 Jun 2025.

[33] Swiss Agency for Therapeutic Products. Swissmedic Annual Report 2022. <https://www.swissmedic.ch/swissmedic/en/home/about-us/publications/aktueller-geschaeftsbericht/swissmedic-geschaeftsbericht-2022.html>. Accessed 3 Jun 2025.

[34] Swiss Agency for Therapeutic Products. Swissmedic Annual Report 2023. <https://www.swissmedic.ch/swissmedic/en/home/about-us/publications/aktueller-geschaeftsbericht/swissmedic-geschaeftsbericht-2023.html>. Accessed 3 Jun 2025.

[35] Global Approved Drug Screening System. Shanghai, China. 2025. <https://webvpn.cpu.edu.cn/https/77726476706e69737468656265737421e6fe51d23e31674a76468aa395/globalapproval/list>. Accessed 3 Jun 2025.

[36] National Healthcare Security Administration. Notice on the issuance of the "National Basic Medical Insurance, Work-related Injury Insurance and Maternity Insurance Drug Catalogue (2023)". <https://www.nhsa.gov.cn/art/2023/12/13/art_104_11673.html>. Accessed 3 Jun 2025.

[37] National Healthcare Security Administration. Notice on the issuance of the "National Basic Medical Insurance, Work-related Injury Insurance and Maternity Insurance Drug Catalogue (2022)". <https://www.nhsa.gov.cn/art/2023/1/18/art_104_10078.html>. Accessed 3 Jun 2025.

[38] National Healthcare Security Administration. Notice on the issuance of the "National Basic Medical Insurance, Work-related Injury Insurance and Maternity Insurance Drug Catalogue (2021)". <https://www.nhsa.gov.cn/art/2021/12/3/art_104_7438.html>. Accessed 3 Jun 2025.

[39] National Healthcare Security Administration. Notice on the issuance of the "National Basic Medical Insurance, Work-related Injury Insurance and Maternity Insurance Drug Catalogue (2020)". <https://www.nhsa.gov.cn/art/2020/12/28/art_53_4223.html>. Accessed 3 Jun 2025.

[40] National Healthcare Security Administration. Notice on the issuance of the "National Basic Medical Insurance, Work-related Injury Insurance and Maternity Insurance Drug Catalogue (2019)". <https://www.nhsa.gov.cn/art/2019/8/20/art_53_1667.html>. Accessed 3 Jun 2025.

[41] National Healthcare Security Administration. Notice on the inclusion of 17 anti-cancer drugs in the Category B of the National Basic Medical Insurance, Work-related Injury Insurance, and Maternity Insurance Drug Catalogue. <https://www.nhsa.gov.cn/art/2018/10/10/art_53_1058.html>. Accessed 3 Jun 2025.

[42] Pharmaceuticals and Medical Devices Agency. Central Social Insurance Medical Council (Central Social Insurance Medical Council General Meeting). <https://www.mhlw.go.jp/stf/shingi/shingi-chuo_128154.html>. Accessed 3 Jun 2025.

[43] Haute Autorité de santé. All publications-Recommendations, medications, procedures, devices, etc. <https://www.has-sante.fr/jcms/fc_2875208/fr/rechercher-une-recommandation-un-avis>. Accessed 3 Jun 2025.

[44] National Institute for Health and Care Excellence. Technology appraisal guidance. <https://www.nice.org.uk/About/What-we-do/Our-Programmes/NICE-guidance/NICE-technology-appraisal-guidance>. Accessed 3 Jun 2025.

[45] Federal Office of Public Health. Spezialitätenliste (SL) und Geburtsgebrechen-Spezialitätenliste (GGSL). <https://www.xn--spezialittenliste-yqb.ch/>. Accessed 3 Jun 2025.

[46] National Medical Products Administration. Center For Drug Evaluation. <https://www.cde.org.cn/main/xxgk/listpage/b40868b5e21c038a6aa8b4319d21b07d>. Accessed 3 Jun 2025.

[47] Pharmaceuticals and Medical Devices Agency. List of products subject to SAKIGAKE designation System and the SAKIGAKE designation System. <https://www.pmda.go.jp/review-services/drug-reviews/0003.html#:~:text=%E5%85%88%E9%A7%86%E3%81%91%E5%AF%A9%E6%9F%BB%E6%8C%87%E5%AE%9A%E5%88%B6%E5%BA%A6%E3%81%AB>. Accessed 3 Jun 2025.

[48] European Medicines Agency. European public assessment report (EPAR). <https://www.ema.europa.eu/en/medicines>. Accessed 3 Jun 2025.

[49] Medicines and Healthcare products Regulatory Agency. Early access to medicines scheme: expired scientific opinions. <https://www.gov.uk/government/publications/early-access-to-medicines-scheme-expired-scientific-opinions>. Accessed 3 Jun 2025.

[50] Pharmaceuticals and Medical Devices Agency. The conditional early approval system for pharmaceuticals. <https://www.mhlw.go.jp/content/10601000/000954228.pdf>. Accessed 3 Jun 2025.

[51] Medicines and Healthcare products Regulatory Agency. Products. <https://products.mhra.gov.uk/>. Accessed 3 Jun 2025.

[52] Swiss Agency for Therapeutic Products. Temporarily authorised human medicines. <https://www.swissmedic.ch/swissmedic/en/home/services/listen_neu.html>. Accessed 3 Jun 2025.

[53] Swiss Agency for Therapeutic Products. Swiss Public Assessment Report (SwissPAR). <https://www.swissmedic.ch/swissmedic/en/home/humanarzneimittel/authorisations/swisspar.html>. Accessed 3 Jun 2025.

[54] National Health Commission of the People's Republic of China. Notice on the releases first catalog of rare diseases. <http://www.nhc.gov.cn/yzygj/s7659/201806/393a9a37f39c4b458d6e830f40a4bb99.shtml>. Accessed 3 Jun 2025.

[55] National Health Commission of the People's Republic of China. Notice on the releases second catalog of rare diseases. <http://www.nhc.gov.cn/yzygj/s7659/202309/19941f5eb0994615b34273bc27bf360d.shtml>. Accessed 3 Jun 2025.

[56] Pharmaceuticals and Medical Devices Agency. List of Designated Drugs for Orphan Diseases. <https://www.nibiohn.go.jp/nibio/part/promote/files/ph_orphanlist_drug_jp.pdf>. Accessed 3 Jun 2025.

[57] European Medicines Agency. Public - List of Opinions on Orphan Medicinal Product Designation. <https://iris.ema.europa.eu/odpublicregister/>. Accessed 3 Jun 2025.

[58] European Medicines Agency. Orphan designations. <https://www.ema.europa.eu/en/medicines/download-medicine-data#orphan-designations-69050>. Accessed 3 Jun 2025.

[59] Medicines and Healthcare products Regulatory Agency. Orphan register. <https://www.gov.uk/government/publications/orphan-registered-medicinal-products/orphan-register>. Accessed 3 Jun 2025.

[60] Swiss Agency for Therapeutic Products. Important medicinal products for rare diseases (orphan drugs in the case of human medicines). <https://www.swissmedic.ch/swissmedic/en/home/services/listen_neu.html>. Accessed 3 Jun 2025.
